# Physical, cognitive, and social triggers of symptom fluctuations in people living with long COVID: an intensive longitudinal cohort study

**DOI:** 10.1016/j.lanepe.2024.101082

**Published:** 2024-09-20

**Authors:** Darren C. Greenwood, Maedeh Mansoubi, Nawar D. Bakerly, Aishwarya Bhatia, Johnny Collett, Helen E. Davies, Joanna Dawes, Brendan Delaney, Leisle Ezekiel, Phaedra Leveridge, Ghazala Mir, Willie Muhlhausen, Clare Rayner, Flo Read, Janet T. Scott, Manoj Sivan, Ian Tucker–Bell, Himanshu Vashisht, Tomás Ward, Daryl B. O'Connor, Helen Dawes, LOCOMOTION consortium, Nawar D. Bakerly, Kumaran Balasundaram, Megan Ball, Mauricio Barahona, Alexander Casson, Jonathan Clarke, Karen Cook, Rowena Cooper, Vasa Curcin, Julie Darbyshire, Helen E. Davies, Helen Dawes, Simon de Lusignan, Brendan Delaney, Carlos Echevarria, Sarah Elkin, Ana Belen Espinosa Gonzalez, Rachael Evans, Sophie Evans, Zacchaeus Falope, Ben Glampson, Madeline Goodwin, Trish Greenhalgh, Darren C. Greenwood, Stephen Halpin, Juliet Harris, Will Hinton, Mike Horton, Samantha Jones, Joseph Kwon, Cassie Lee, Ashliegh Lovett, Mae Mansoubi, Victoria Masey, Harsha Master, Erik Mayer, Bernardo Meza-Torres, Ruairidh Milne, Ghazala Mir, Jacqui Morris, Adam Mosley, Jordan Mullard, Daryl O'Connor, Rory O'Connor, Thomas Osborne, Amy Parkin, Stavros Petrou, Anton Pick, Denys Prociuk, Clare Rayner, Amy Rebane, Natalie Rogers, Janet T. Scott, Manoj Sivan, Adam B. Smith, Nikki Smith, Emma Tucker, Ian Tucker-Bell, Paul Williams, Darren Winch, Conor Wood

**Affiliations:** a School of Medicine, University of Leeds, Leeds, UK; b Leeds Institute for Data Analytics, University of Leeds, Leeds, UK; c NIHR Exeter Biomedical Research Centre, University of Exeter, Exeter, UK; d Medical School, University of Exeter, Exeter, UK; e Department of Public Health and Sport Sciences, University of Exeter, Exeter, UK; f Northern Care Alliance, Salford, UK; g Department of Sport, Health and Social Work, Oxford Brookes University, Oxford, UK; h Cardiff and Vale University Health Board, Cardiff, UK; i University Hospitals Birmingham NHS Foundation Trust, Birmingham, UK; j Faculty of Medicine, Department of Surgery & Cancer, Imperial College, London, UK; k School of Health Sciences, University of Southampton, Southampton, UK; l Leeds Institute of Health Sciences, University of Leeds, Leeds, UK; m In the Wild Research Limited, Dublin, Ireland; n Patient Advisory Group (PAG) Representative, UK; o Department of Health and Community Sciences, University of Exeter, Exeter, UK; p COVID Recovery Service, NHS Highlands, Raigmore Hospital, Inverness, UK; q MRC-University of Glasgow Centre for Virus Research, Glasgow, UK; r Academic Department of Rehabilitation Medicine, University of Leeds, Leeds, UK; s Insight SFI Research Centre for Data Analytics, Dublin City University, Dublin, Ireland; t School of Psychology, University of Leeds, Leeds, UK

**Keywords:** Long COVID, Post-acute COVID-19 syndrome, Physical exertion, Mental exertion, Symptoms, Ecological momentary analysis, Intensive longitudinal methods

## Abstract

**Background:**

Symptom fluctuations within and between individuals with long COVID are widely reported, but the extent to which severity varies following different types of activity and levels of exertion, and the timing of symptoms and recovery, have not previously been quantified. We aimed to characterise timing, severity, and nature of symptom fluctuations in response to effortful physical, social and cognitive activities, using Ecological Momentary Assessments.

**Methods:**

We recorded activity, effort, and severity of 8 core symptoms every 3 h for up to 24 days, in cohorts from both clinic and community settings. Symptom severities were jointly modelled using autoregressive and moving average processes.

**Findings:**

Consent was received from 376 participants providing ≥1 week's measurements (273 clinic-based, 103 community-based). Severity of all symptoms was elevated 30 min after all categories of activity. Increased effort was associated with increased symptom severity. Fatigue severity scores increased by 1.8/10 (95% CI: 1.6–1.9) following the highest physical exertions and by 1.5 (1.4–1.7) following cognitive efforts. There was evidence of only mild delayed fatigue 3 h (0.3, 0.2–0.5) or one day later (0.2, 0.0– 0.5). Fatigue severity increased as the day progressed (1.4, 1.0–1.7), and cognitive dysfunction was 0.2 lower at weekends (0.1–0.3).

**Interpretation:**

Cognitive, social, self-care and physical activities all triggered increased severity across every symptom, consistent with associated common pathways as potential therapeutic targets. Clear patterns of symptom fluctuations emerged that support more targeted self-management.

**Funding:**

10.13039/501100000272National Institute for Health and Care Research.

**Research in context**

**Evidence before this study**

We searched Medline using search terms “Post-Acute COVID-19 Syndrome” and “physical exertion” or “cognitive exertion”, including MeSH terms and synonyms, between 1st January 2020 and 31st March 2021, restricted to English language. We found no previous intensive longitudinal cohorts examining exertion and subsequent symptom severity. One after that date only investigated physical activity and fatigue. Most evidence for delayed symptom response was largely anecdotal.

**Added value of this study**

The present study is the first to quantify the immediate time of the activity, and delayed increases later that day or the following day. We have shown that these associations are much the same for physical and cognitive exertions, and extend to effortful social and self-care activities.

**Implications of all the available evidence**

Our findings are consistent with current symptom management advice, but underline the importance of managing all types of effortful activity, not just physical. The wide range of symptoms with associated increased severity suggest common pathways as potential therapeutic targets.

## Introduction

An estimated 10% of COVID-19 survivors continue to report symptoms persisting past 12 weeks.^1^, ^2^, ^3^ Long COVID and post-COVID-19 syndrome is not restricted to people who experienced a severe acute infection or those requiring hospital admission.^4^ Reported symptoms can be wide ranging but frequently include fatigue, breathlessness, palpitations, dizziness, pain, cognitive dysfunction, anxiety and depression,^1^^,^^2^ which may fluctuate across the day and between successive days within the same individual.^5^, ^6^, ^7^ A characteristic aspect of many patients’ experience of long COVID is the marked and often unpredictable variation of symptoms that occurs over periods of hours and days and the heterogeneity of response to and recovery from potential triggering events.^5^^,^^8^ Rest or sleep during the day, or quality of sleep at night, may offer mitigation against the effects of earlier exertions, but not for all.^6^^,^^9^ There is no consensus on mechanism or established treatment for managing long COVID symptoms.

Potential actions that may trigger symptoms include physical, cognitive, social or emotional effortful activities. Self-care activities such as taking a shower or cooking a meal have also been reported to provide a trigger. To date, research has focused on physical activities, or other triggers in isolation.^10^ Understanding the nature and timing of events that trigger subsequent reoccurrence or increased severity of symptoms, and the profile of that response, may enable individuals to better manage their long COVID and facilitate more targeted intervention.^11^^,^^12^ Whilst immune system dysregulation is likely involved, there is limited understanding of the underlying pathology, mechanisms and pathways to symptom presentation, and a subsequent lack of consensus on treatment beyond symptom management.^13^^,^^14^

Our aim was to identify underlying routes of symptom response to activities, to inform more targeted intervention for symptom management for people living with long COVID. Utilising smartphones to apply intensive longitudinal methods,^15^^,^^16^ we characterised how different symptoms fluctuate in response to different types of triggering activities throughout the day, over three 8-day assessment periods, each separated by several weeks, in a large cohort of individuals living with long COVID.

## Methods

### Design and setting

This intensive longitudinal cohort study forms part of the long COVID Multi-Disciplinary Consortium to Optimise Treatments and Services across the NHS (LOCOMOTION) research programme.^17^ LOCOMOTION is a multisite programme, including technology-supported monitoring of condition-specific outcome measures, registered with ISRCTN (ISRCTN15022307). A detailed protocol for the current work has been published previously,^18^ and is summarised here focusing on our primary objective of quantifying the extent to which activities predict subsequent symptoms using Ecological Momentary Assessments (EMAs).

Eligible participants were aged 18 years and over, recruited across ten long COVID services within the UK National Health Service participating in the LOCOMOTION consortium between February 2022 and August 2023, irrespective of whether they were hospitalised or had a positive or negative SARS-CoV-2 test.^17^^,^^18^ An additional sample was recruited in the community through general practice networks and social media. Exclusion criteria were inability to use mobile phone or wearable technology or understand the language used, known pregnancy, or known previous diagnosis of dementia or cognitive impairment.

Participants provided demographic information on recruitment including age, sex, ethnicity, employment, infection history, vaccination history. They then completed EMAs at five time points spread over the day, on eight consecutive days. This was then repeated for a further eight consecutive days, at six and then 12 weeks after recruitment (Supplemental Figure S1). Participants with less than one week's EMA data were excluded. Study data were collected and managed using REDCap electronic data capture tools hosted at the University of Exeter^19^ and the EMA delivered using the AthenaCX platform for mobile phones.^20^

### Ecological momentary assessments

Participants were notified of each EMA through push notifications on their phone, with responses permitted within a 45-min window. The EMA was co-designed with long COVID patients, based on previous work.^8^^,^^18^ Usability and acceptability were determined in a proof-of-concept phase. Each EMA collected details of the dominant activity (physical, cognitive, social, self-care, rest or sleep) during the preceding 30 min, the amount of effort associated with the activity (scored 0 “no effort” to 10 “most effortful”), presence of current symptoms and severity (scored 0 “no problem” to 10 “severe problem”). The list of core symptoms was adapted from the COVID-19 Yorkshire Rehabilitation Scale (C19-YRS) items,^21^ including fatigue, pain or discomfort, dizziness, palpitations, cognitive dysfunction, anxiety and depression. At the start of each day, participants were asked about sleep quality the night before. Example EMA screenshots are shown in Supplemental Figure S2.

### Sample size

Based on data from service evaluation conducted within a COVID Rehabilitation service in the North of England,^22^ we anticipated 300 participants would provide approximately 80% power to detect a 20% improvement in fatigue over 12 weeks in one of three equally sized groups of participants relative to another. For example, a group with fatigue severity scores of 5 at each timepoint, compared with a group gradually improving from 5 to 4, assuming a within-person correlation of 0.7 and a residual variance 2.0, taking account of repeated measures on successive days. We anticipated up to 25% dropout across follow-up, so aimed to recruit 400 individuals to the study.

### Ethical considerations

Ethical approval was provided by the Yorkshire & The Humber–Bradford Leeds Research Ethics Committee (ref: 21/YH/0276). All participants provided written informed consent to participate, and the research conforms to the Declaration of Helsinki.

### Statistical analysis

Models quantifying the associations between the activities and subsequent symptom scores took account of the hierarchical data structure of times, within days, within participants, with symptom scores as joint multivariate outcomes. The best fitting model incorporated time dependency between symptoms was incorporated using a first-order moving average (MA1) process at both time and day level. Associations between activity efforts and symptoms used an autoregressive time-series of order 1 (AR1).

All models included person-level covariates: age, sex, ethnicity, employment status, location (clinic or community setting), acute infection severity, whether hospitalised, admitted to intensive care unit, dominant variant at infection, vaccination status, duration of long COVID. Models also included time-level covariates: time of day and activity exertions, and day-level covariates: rest during the day, sleep during the day, overnight sleep quality. Absence of a reported activity was assumed to imply zero exertion on that activity. Missing response data were assumed missing at random (MAR) with values generated from the posterior predictive distribution.

Confidence intervals for prevalence of symptoms and mean severity scores were derived from simple variance-components models allowing for variation at person, week, day and time-levels using Stata (version 18). Models of associations and time dependencies used Markov chain Monte Carlo (MCMC) methods in JAGS 4.3.0 using the runjags package from within R (version 4.3.1) on the High Performance Computing facilities at the University of Leeds, UK. Credible intervals (95%) and two-sided p-values were derived. Additional statistical methods are available in Supplemental Methods and Supplemental Table S1.

### Subgroup analyses

To explore potential differences between predefined subgroups, the AR1 model using mean activity efforts was fitted separately for clinic and community samples, for men and women, and for younger (<50 years) and older participants (50+ years).

### Patient and public involvement

This study was co-designed with people living with long COVID, ensuring aims, objectives, questionnaires, recruitment and dissemination of findings reflect the priorities of those living with long COVID. The LOCOMOTION study has a seven-member core Patient and public involvement (PPI) advisory group, which aimed to include different cultural, ethnic and socioeconomic groups. Three members trialled study methods and provided feedback on wording and implementation to ensure EMAs were straightforward and minimum burden to participants. Two members of this group are co-authors of this work.

### Role of the funding source

The funder had no role in study design, data collection, analysis, interpretation, in the writing the report or the decision to submit for publication.

## Results

### Recruitment

In total, 514 participants were approached (351 from clinics, 163 community), and of these 420 (82%) participants consented to take part in the daily monitoring of symptoms (301 from clinics, 119 from the community), with 376 (73%) providing at least one week's EMA data (273 from clinics, 103 community) with a median adherence rate of 75%. Mean (SD) age of participants was 47 (11) years, with 274 (73%) female. 335 (89%) reported a positive COVID-19 test. Participant characteristics are shown in Supplemental Table S2.

### Descriptive statistics

Fatigue was the most frequently reported symptom of the EMA measures (83% of the time), followed by cognitive dysfunction (47%) and pain or discomfort (39%) (Table 1). Participants aged 60+ years reported breathlessness approximately twice as often as <40 years. Women reported pain or discomfort nearly twice as often as men. Participants who were admitted to hospital or ICU with initial infection experienced more frequent breathlessness, fatigue, anxiety and cognitive dysfunction. Participants able to work full-time reported lower frequency and less severe symptoms than those unable to work full-time. Participants recruited in the community reported similar frequency and severity of most symptoms as participants long COVID clinics, with fatigue more frequently reported (Table 1, Table 2).

**Table 1:** Observed prevalence of symptoms at any one time, by participant characteristics, with 95% confidence intervals.

	Breathless-ness	Fatigue	Pain/discomfort	Dizziness	Palpitations	Anxiety	Depression	Cognitive dysfunction
All participants	19% (16, 21)	83% (80, 86)	39% (34, 44)	6% (5, 7)	4% (3, 5)	21% (18, 24)	5% (4, 7)	47% (43, 52)
Age (years):								
<40	16% (12, 21)	79% (73, 85)	38% (30, 48)	8% (6, 11)	5% (4, 7)	19% (14, 27)	8% (5, 12)	40% (33, 50)
40–49	18% (14, 23)	83% (78, 89)	42% (34, 52)	6% (4, 8)	4% (3, 6)	19% (15, 25)	5% (3, 7)	53% (45, 62)
50–59	19% (15, 24)	84% (80, 89)	38% (31, 47)	5% (4, 7)	4% (3, 5)	22% (16, 28)	4% (3, 7)	49% (42, 59)
60+	30% (21, 43)	88% (82, 94)	33% (21, 51)	6% (3, 12)	2% (1, 5)	26% (18, 36)	4% (2, 8)	43% (31, 61)
Gender:								
Female	18% (16, 22)	87% (85, 90)	45% (40, 51)	6% (5, 8)	5% (4, 6)	20% (17, 24)	5% (4, 6)	46% (41, 52)
Male	19% (14, 25)	73% (65, 81)	25% (18, 34)	6% (4, 9)	3% (2, 4)	22% (16, 31)	6% (4, 10)	49% (40, 60)
Ethnicity:								
White ethnicity	19% (16, 22)	83% (80, 86)	37% (33, 43)	6% (5, 8)	4% (3, 5)	21% (18, 24)	5% (4, 6)	48% (43, 54)
Minority ethnic	17% (11, 25)	87% (81, 93)	48% (37, 62)	5% (3, 9)	5% (3, 9)	21% (13, 33)	7% (4, 13)	40% (30, 54)
Employment status:								
Full-time	18% (15, 22)	75% (69, 81)	32% (26, 39)	6% (4, 8)	3% (2, 4)	18% (14, 23)	4% (3, 6)	40% (34, 48)
Part-time	17% (13, 23)	89% (86, 92)	38% (30, 48)	5% (3, 8)	4% (2, 5)	23% (18, 31)	5% (3, 9)	56% (48, 64)
Other	21% (16, 26)	91% (88, 94)	53% (44, 63)	8% (5, 11)	6% (4, 9)	22% (17, 29)	7% (5, 11)	52% (43, 62)
Source of participants:								
Clinics	19% (16, 22)	79% (75, 83)	36% (31, 42)	6% (4, 7)	3% (3, 4)	21% (18, 25)	5% (4, 7)	44% (39, 50)
Community	18% (14, 24)	94% (92, 96)	46% (37, 57)	8% (5, 11)	6% (4, 9)	20% (15, 26)	5% (3, 8)	55% (46, 65)
Vaccination status at time of infection:								
2+ courses	18% (15, 22)	85% (82, 89)	36% (31, 43)	6% (5, 8)	3% (2, 4)	25% (21, 30)	6% (4, 8)	50% (44, 56)
<2 courses	19% (15, 23)	80% (75, 85)	42% (35, 50)	6% (5, 8)	5% (4, 7)	17% (13, 21)	4% (3, 6)	44% (37, 52)
Dominant variant at time of infection:								
Original	18% (14, 23)	83% (78, 89)	39% (31, 49)	7% (5, 9)	5% (3, 7)	17% (13, 23)	3% (2, 5)	46% (38, 56)
Alpha	29% (20, 40)	78% (67, 91)	41% (28, 60)	6% (3, 13)	8% (4, 13)	19% (12, 32)	5% (2, 11)	50% (37, 67)
Delta	19% (14, 24)	83% (77, 90)	40% (32, 52)	6% (4, 9)	4% (2, 5)	21% (16, 30)	5% (3, 9)	58% (49, 67)
Omicron	17% (13, 22)	84% (80, 89)	37% (30, 45)	6% (4, 8)	3% (2, 4)	25% (20, 31)	7% (5, 11)	41% (34, 49)
Severity of initial infection:								
Asymptomatic	21% (12, 37)	60% (36, 100)	44% (23, 83)	5% (1, 16)	5% (1, 18)	6% (2, 22)	4% (1, 15)	36% (15, 87)
Admitted to hospital	26% (18, 36)	79% (67, 93)	32% (20, 50)	4% (2, 7)	4% (2, 8)	21% (12, 38)	8% (4, 16)	46% (33, 65)
Admitted to ICU	30% (18, 50)	93% (87, 100)	36% (17, 76)	3% (1, 9)	1% (0, 8)	42% (24, 74)	8% (3, 27)	52% (29, 95)
Duration of long COVID (years):								
<1	17% (13, 22)	80% (75, 86)	30% (23, 38)	4% (3, 6)	2% (2, 4)	23% (18, 29)	5% (3, 8)	39% (32, 47)
1+	19% (17, 23)	85% (81, 88)	44% (38, 51)	7% (6, 9)	5% (4, 7)	20% (16, 24)	5% (4, 7)	52% (47, 58)

**Table 2:** Mean observed symptom severity scores (0–10), by participant characteristics, with 95% confidence intervals.

	Breathless-ness	Fatigue	Pain/discomfort	Dizziness	Palpitations	Anxiety	Depression	Cognitive dysfunction
All participants	1.3 (1.1, 1.4)	4.9 (4.7, 5.1)	2.9 (2.7, 3.2)	0.9 (0.7, 1.0)	0.6 (0.5, 0.7)	1.9 (1.7, 2.1)	1.2 (1.0, 1.4)	3.2 (2.9, 3.4)
Age (years):								
<40	1.0 (0.7, 1.3)	4.7 (4.2, 5.1)	2.6 (2.2, 3.1)	0.8 (0.5, 1.1)	0.5 (0.3, 0.6)	1.7 (1.4, 2.1)	1.3 (0.9, 1.7)	2.7 (2.2, 3.1)
40–49	1.2 (1.0, 1.5)	4.9 (4.5, 5.3)	3.1 (2.7, 3.6)	0.8 (0.6, 1.1)	0.6 (0.4, 0.8)	1.8 (1.4, 2.2)	1.3 (0.9, 1.7)	3.4 (3.0, 3.8)
50–59	1.3 (1.0, 1.6)	5.1 (4.7, 5.4)	3.0 (2.5, 3.4)	0.8 (0.5, 1.0)	0.6 (0.4, 0.8)	2.1 (1.7, 2.5)	1.4 (1.0, 1.7)	3.3 (2.9, 3.7)
60+	2.0 (1.4, 2.5)	5.2 (4.7, 5.8)	2.9 (2.2, 3.6)	1.2 (0.7, 1.7)	0.6 (0.3, 1.0)	1.8 (1.2, 2.3)	0.7 (0.3, 1.2)	3.3 (2.6, 4.1)
Gender:								
Female	1.2 (1.0, 1.3)	5.1 (4.9, 5.3)	3.1 (2.8, 3.3)	0.8 (0.6, 0.9)	0.5 (0.4, 0.7)	1.7 (1.5, 2.0)	1.0 (0.8, 1.3)	3.1 (2.8, 3.4)
Male	1.5 (1.2, 1.9)	4.5 (4.0, 4.9)	2.5 (2.0, 3.0)	1.0 (0.7, 1.4)	0.6 (0.4, 0.9)	2.3 (1.8, 2.8)	1.8 (1.3, 2.2)	3.4 (2.9, 3.9)
Ethnicity:								
White ethnicity	1.3 (1.1, 1.4)	4.9 (4.7, 5.1)	2.8 (2.6, 3.1)	0.8 (0.7, 1.0)	0.6 (0.4, 0.7)	1.8 (1.6, 2.1)	1.2 (1.0, 1.4)	3.2 (2.9, 3.4)
Minority ethnic	1.4 (0.9, 1.8)	5.3 (4.7, 5.8)	3.5 (2.8, 4.3)	1.0 (0.5, 1.5)	0.7 (0.4, 1.0)	2.3 (1.6, 2.9)	1.4 (0.8, 2.0)	3.3 (2.5, 4.0)
Employment status:								
Full-time	1.2 (1.0, 1.4)	4.6 (4.3, 4.9)	2.6 (2.3, 3.0)	0.8 (0.6, 1.0)	0.5 (0.3, 0.6)	1.9 (1.6, 2.2)	1.1 (0.9, 1.4)	2.9 (2.5, 3.3)
Part-time	1.2 (0.9, 1.5)	4.9 (4.5, 5.2)	2.7 (2.3, 3.2)	0.6 (0.4, 0.8)	0.5 (0.3, 0.7)	1.8 (1.4, 2.2)	1.1 (0.8, 1.5)	3.1 (2.7, 3.6)
Other	1.5 (1.2, 1.8)	5.5 (5.1, 5.8)	3.6 (3.1, 4.0)	1.1 (0.8, 1.4)	0.8 (0.5, 1.0)	1.9 (1.6, 2.3)	1.5 (1.1, 1.9)	3.6 (3.1, 4.1)
Source of participants:								
Clinics	1.3 (1.1, 1.5)	4.7 (4.5, 5.0)	2.9 (2.6, 3.2)	0.8 (0.6, 0.9)	0.5 (0.4, 0.6)	1.9 (1.7, 2.2)	1.3 (1.1, 1.6)	3.0 (2.7, 3.3)
Community	1.2 (1.0, 1.5)	5.5 (5.1, 5.8)	3.1 (2.6, 3.5)	1.1 (0.8, 1.4)	0.7 (0.5, 0.9)	1.8 (1.4, 2.2)	1.1 (0.7, 1.4)	3.6 (3.1, 4.0)
Vaccination status at time of infection:								
2+ courses	1.2 (1.0, 1.4)	4.8 (4.5, 5.1)	2.7 (2.3, 3.0)	0.8 (0.6, 1.0)	0.5 (0.3, 0.6)	1.9 (1.7, 2.2)	1.1 (0.9, 1.4)	3.1 (2.7, 3.4)
<2 courses	1.4 (1.2, 1.6)	5.1 (4.7, 5.4)	3.2 (2.8, 3.6)	0.9 (0.7, 1.1)	0.7 (0.5, 0.9)	1.8 (1.5, 2.1)	1.4 (1.1, 1.7)	3.3 (2.9, 3.7)
Dominant variant at time of infection:								
Original	1.3 (1.0, 1.5)	5.0 (4.7, 5.4)	3.1 (2.7, 3.5)	0.8 (0.6, 1.0)	0.7 (0.5, 0.9)	1.7 (1.4, 2.0)	1.2 (0.8, 1.5)	3.3 (2.8, 3.7)
Alpha	2.1 (1.4, 2.8)	5.4 (4.6, 6.3)	3.6 (2.6, 4.6)	1.4 (0.7, 2.2)	1.1 (0.5, 1.7)	2.5 (1.5, 3.5)	1.9 (1.0, 2.9)	3.9 (3.0, 4.9)
Delta	1.2 (1.0, 1.5)	4.9 (4.5, 5.4)	3.1 (2.6, 3.6)	0.8 (0.5, 1.1)	0.4 (0.3, 0.6)	2.0 (1.5, 2.4)	1.1 (0.8, 1.5)	3.4 (2.9, 3.9)
Omicron	1.1 (0.9, 1.4)	4.7 (4.4, 5.1)	2.5 (2.1, 2.8)	0.8 (0.6, 1.0)	0.4 (0.3, 0.6)	1.9 (1.5, 2.2)	1.2 (0.9, 1.6)	2.7 (2.3, 3.2)
Severity of initial infection:								
Asymptomatic	0.9 (0.4, 1.4)	4.2 (2.9, 5.6)	3.1 (1.8, 4.3)	0.6 (0.1, 1.2)	0.9 (0.1, 1.8)	1.3 (0.2, 2.5)	1.0 (0.1, 1.9)	2.7 (1.5, 3.9)
Admitted to hospital	1.5 (0.9, 2.1)	5.0 (4.2, 5.8)	2.7 (1.9, 3.5)	0.7 (0.2, 1.2)	0.8 (0.2, 1.3)	2.3 (1.5, 3.1)	1.6 (0.8, 2.4)	3.1 (2.3, 3.9)
Admitted to ICU	1.9 (0.8, 3.1)	5.6 (4.6, 6.6)	3.2 (1.5, 4.9)	0.8 (0, 2.1)	1.0 (0, 2.3)	3.2 (1.6, 4.8)	2.0 (0.3, 3.6)	3.8 (2.3, 5.4)
Duration of long COVID (years):								
<1	1.1 (0.9, 1.4)	4.6 (4.2, 4.9)	2.3 (1.9, 2.7)	0.7 (0.4, 0.9)	0.4 (0.3, 0.6)	1.9 (1.5, 2.2)	1.2 (0.9, 1.5)	2.7 (2.3, 3.1)
1+	1.4 (1.2, 1.6)	5.1 (4.8, 5.4)	3.2 (2.9, 3.5)	1.0 (0.8, 1.1)	0.6 (0.5, 0.8)	1.9 (1.6, 2.1)	1.3 (1.0, 1.5)	3.4 (3.1, 3.7)

### Participant characteristics as predictors of symptom severity

Table 3 shows the estimated difference in mean symptom severity scores (rated 0–10) for each participant characteristic, with 95% credible intervals, adjusted for covariates. Older age was a significant predictor of breathlessness (0.7 for 60+ years vs <40, 0.3–.1, p = 0.01), with pain or discomfort and cognitive dysfunction most felt in participants aged 40 to 59. Women had more fatigue than men (difference = 0.5, 95% CI: 0.1–1.0, p = 0.02), but less dizziness (−0.4, −0.7 to 0.0, p = 0.03), anxiety (−0.7, −1.1 to −0.2, p = 0.007) and depression (−0.9, −1.3 to −0.4, p < 0.001) than men. There was no evidence that minority ethnic groups as a whole experienced different symptom severity than white ethnicity. Participants who were able to stay in full-time employment had less fatigue (−0.6, −1.1 to −0.2, p = 0.007), pain/discomfort (−1.0, −1.6 to −0.5, p < 0.001), depression (−0.6, −1.1 to −0.1, p = 0.02) and cognitive dysfunction (−0.7, −1.3 to −0.1, p = 0.02). Participants recruited from the community reported more fatigue than those recruited from long COVID clinics (0.5, 0.0 to 0.9, p = 0.04), but there was no evidence of difference in long COVID symptom severities between those whose initial infection was asymptomatic, admitted to hospital, or ICU. Participants with longer duration of long COVID symptoms tended to have more pain or discomfort (0.8 *per* year, 0.2–1.3, p = 0.004).

**Table 3:** Difference in mean symptom severity scores between participant characteristics, adjusted for covariates, with 95% credible intervals.

	Breathless-ness	Fatigue	Pain/discomfort	Dizziness	Palpitations	Anxiety	Depression	Cognitive dysfunction
**Person-level characteristics**^a^								
Age (years):								
40–49 vs <40	0.2 (−0.1, 0.5)	0.4 (0.0, 0.8)	0.9 (0.4, 1.3)	0.1 (−0.2, 0.4)	0.1 (−0.1, 0.4)	0.4 (0.0, 0.8)	0.2 (0.0, 0.6)	0.6 (0.1, 1.1)
50–59 vs <40	0.4 (0.1, 0.7)	0.5 (0.0, 0.9)	0.7 (0.2, 1.2)	0.1 (−0.2, 0.5)	0.1 (−0.2, 0.4)	0.4 (−0.1, 0.9)	0.0 (−0.5, 0.5)	0.6 (0.0, 1.1)
60+ vs < 40	0.7 (0.3, 1.1)	0.5 (−0.1, 1.1)	0.0 (−0.7, 0.7)	0.1 (−0.3, 0.6)	0.0 (−0.4, 0.3)	0.2 (−0.4, 0.9)	−0.5 (−1.1, 0.2)	0.2 (−0.6, 0.9)
Gender:								
Male vs female	0.3 (0.0, 0.7)	−0.5 (−1.0, −0.1)	−0.4 (−0.9, 0.2)	0.4 (0.0, 0.7)	0.1 (−0.1, 0.4)	0.7 (0.2, 1.1)	0.9 (0.4, 1.3)	0.5 (−0.1, 1.0)
Ethnicity:								
Minority ethnic vs White	0.1 (−0.3, 0.6)	0.4 (−0.2, 1.0)	0.7 (0.0, 1.4)	0.3 (−0.2, 0.7)	0.2 (−0.2, 0.5)	0.4 (−0.2, 1.0)	0.2 (−0.4, 0.8)	0.1 (−0.6, 0.8)
Employment status:								
Full-time vs none	−0.2 (−0.5, 0.2)	−0.6 (−1.1, −0.2)	−1.0 (−1.6, −0.5)	−0.3 (−0.6, 0.1)	−0.2 (−0.5, 0.0)	−0.3 (−0.8, 0.2)	−0.6 (−1.1, −0.1)	−0.7 (−1.3, −0.1)
Part-time vs none	−0.2 (−0.6, 0.2)	−0.6 (−1.1, 0.0)	−1.1 (−1.8, −0.5)	−0.5 (−0.9, 0.0)	−0.3 (−0.6, 0.0)	−0.4 (−0.9, 0.2)	−0.5 (−1.0, 0.1)	−0.6 (−1.3, 0.1)
Source of participants:								
Community vs clinics	−0.2 (−0.5, 0.2)	0.5 (0.0, 0.9)	−0.4 (−1.0, 0.1)	0.3 (−0.1, 0.7)	0.1 (−0.2, 0.4)	−0.2 (−0.7, 0.3)	−0.3 (−0.8, 0.2)	0.2 (−0.4, 0.8)
Vaccination status at time of infection:								
2+ courses vs not	0.1 (−0.4, 0.6)	0.0 (−0.7, 0.7)	−0.1 (−0.9, 0.8)	0.2 (−0.4, 0.7)	0.1 (−0.3, 0.5)	0.4 (−0.4, 1.1)	−0.2 (−1.0, 0.5)	0.7 (−0.2, 1.5)
Dominant variant at time of infection:								
Alpha vs original	0.8 (0.2, 1.4)	0.4 (−0.4, 1.2)	1.2 (0.3, 2.1)	0.8 (0.2, 1.4)	0.5 (0.0, 0.9)	0.7 (−0.1, 1.5)	0.6 (−0.2, 1.4)	1.1 (0.1, 2.0)
Delta vs original	0.0 (−0.6, 0.6)	0.2 (−0.6, 1.0)	1.1 (0.2, 2.1)	0.1 (−0.6, 0.7)	−0.1 (−0.6, 0.3)	0.1 (−0.8, 0.9)	0.0 (−0.8, 0.8)	0.3 (−0.7, 1.3)
Omicron vs original	0.0 (−0.7, 0.7)	0.0 (−0.9, 0.9)	0.9 (−0.2, 2.0)	0.1 (−0.7, 0.8)	0.0 (−0.6, 0.5)	0.1 (−0.9, 1.1)	0.3 (−0.7, 1.3)	−0.2 (−1.4, 0.9)
Severity of initial infection:								
Asymptomatic vs not	−0.4 (−1.2, 0.5)	−0.2 (−1.3, 0.9)	0.6 (−0.8, 1.9)	−0.2 (−1.1, 0.7)	0.4 (−0.2, 1.1)	−0.4 (−1.6, 0.8)	−0.4 (−1.6, 0.8)	−0.2 (−1.5, 1.2)
Admitted to hospital vs not	0.0 (−0.6, 0.6)	−0.1 (−0.9, 0.7)	−0.6 (−1.5, 0.4)	−0.3 (−0.9, 0.4)	0.1 (−0.4, 0.6)	0.1 (−0.8, 0.9)	0.1 (−0.7, 1.0)	−0.5 (−1.5, 0.6)
Admitted to ICU vs not	0.3 (−0.6, 1.1)	0.5 (−0.6, 1.7)	0.2 (−1.2, 1.6)	−0.2 (−1.1, 0.7)	0.4 (−0.2, 1.1)	0.8 (−0.4, 2.0)	0.2 (−1.0, 1.4)	0.2 (−1.2, 1.6)
Duration of long COVID (per year):	0.1 (−0.2, 0.4)	0.1 (−0.4, 0.5)	0.8 (0.2, 1.3)	0.1 (−0.2, 0.5)	0.1 (−0.1, 0.4)	0.1 (−0.4, 0.6)	0.0 (−0.5, 0.4)	0.5 (0.0, 1.1)
**Day-level characteristics**^b^								
Longer-term trend (per month):	0.0 (−0.1, 0.0)	0.0 (−0.1, 0.0)	0.0 (0.0, 0.1)	0.0 (−0.1, 0.0)	0.0 (0.0, 0.0)	0.0 (−0.1, 0.0)	0.0 (0.0, 0.1)	0.0 (0.0, 0.0)
Day of week:								
Weekday vs weekend	0.0 (0.0, 0.1)	0.2 (0.1, 0.3)	0.1 (0.1, 0.2)	0.1 (0.0, 0.1)	0.0 (0.0, 0.1)	0.1 (0.1, 0.2)	0.1 (0.0, 0.1)	0.3 (0.2, 0.3)
Quality of sleep (0–10):								
Bad (<3) vs good (5–6.9)	0.1 (0.0, 0.2)	0.3 (0.2, 0.4)	0.1 (0.0, 0.3)	0.1 (0.0, 0.1)	0.0 (−0.1, 0.1)	0.1 (0.0, 0.2)	0.1 (0.0, 0.1)	0.1 (0.0, 0.3)
Poor (3–4.9) vs good (5–6.9)	0.1 (0.0, 0.1)	0.1 (0.1, 0.2)	0.1 (0.0, 0.2)	0.0 (−0.1, 0.1)	0.0 (0.0, 0.1)	0.1 (0.0, 0.1)	0.0 (−0.1, 0.1)	0.0 (−0.1, 0.1)
Great (7+) vs good (5–6.9)	0.0 (−0.1, 0.1)	−0.1 (−0.2, 0.0)	−0.1 (−0.2, 0.0)	−0.1 (−0.1, 0.0)	0.0 (−0.1, 0.1)	−0.1 (−0.2, 0.0)	−0.1 (−0.1, 0.0)	−0.1 (−0.2, 0.0)
Not reported vs good (5–6.9)	0.1 (0.0, 0.2)	0.2 (0.1, 0.3)	0.1 (0.0, 0.2)	0.1 (0.0, 0.1)	0.1 (0.0, 0.1)	0.1 (0.0, 0.2)	0.1 (0.0, 0.1)	0.1 (0.0, 0.2)
**Time-level characteristics**^c^								
Time of day:								
12 pm vs 9 am	0.0 (−0.1, 0.1)	0.3 (0.2, 0.4)	0.0 (0.0, 0.1)	0.1 (0.0, 0.1)	0.0 (−0.1, 0.1)	−0.1 (−0.2, 0.0)	0.0 (0.0, 0.1)	0.2 (0.1, 0.3)
3 pm vs 9 am	0.0 (−0.1, 0.1)	0.7 (0.5, 0.9)	0.1 (−0.1, 0.2)	0.2 (0.1, 0.3)	0.0 (−0.1, 0.1)	−0.2 (−0.3, 0.0)	0.0 (−0.1, 0.2)	0.4 (0.2, 0.5)
6 pm vs 9 am	0.0 (−0.2, 0.2)	1.1 (0.7, 1.3)	0.1 (−0.1, 0.4)	0.3 (0.1, 0.4)	0.0 (−0.2, 0.2)	−0.2 (−0.5, 0.0)	0.0 (−0.1, 0.2)	0.6 (0.3, 0.8)
9 pm vs 9 am	0.0 (−0.3, 0.3)	1.4 (1.0, 1.7)	0.2 (−0.1, 0.5)	0.3 (0.1, 0.6)	0.0 (−0.3, 0.2)	−0.3 (−0.6, 0.0)	0.1 (−0.1, 0.3)	0.7 (0.4, 1.1)
Sleep and rest:								
Rest 30 min before vs not	0.1 (0.0, 0.2)	0.6 (0.5, 0.7)	0.2 (0.1, 0.3)	0.0 (0.0, 0.1)	0.0 (0.0, 0.1)	0.1 (0.0, 0.2)	0.1 (0.0, 0.1)	0.2 (0.1, 0.3)
Sleep 30 min before vs not	0.0 (−0.1, 0.2)	1.0 (0.9, 1.2)	0.3 (0.2, 0.5)	−0.1 (−0.2, 0.1)	0.0 (−0.1, 0.1)	−0.1 (−0.2, 0.1)	0.0 (−0.1, 0.1)	−0.1 (−0.2, 0.1)
Rest 3 h before vs not	0.0 (−0.1, 0.1)	0.0 (0.0, 0.1)	0.1 (0.0, 0.1)	0.0 (0.0, 0.1)	0.0 (−0.1, 0.0)	0.0 (−0.1, 0.1)	0.0 (0.0, 0.1)	−0.1 (−0.2, 0.0)
Sleep 3 h before vs not	−0.1 (−0.2, 0.0)	0.1 (0.0, 0.2)	0.1 (0.0, 0.2)	0.0 (−0.1, 0.1)	0.0 (−0.1, 0.0)	0.0 (−0.1, 0.1)	0.1 (0.0, 0.2)	−0.1 (−0.2, 0.0)

Estimates present difference in mean symptom score (rated 0 to 10) compared to reference group, adjusted for covariates at the person-level, day-level and time-level.

a Person-level covariates: age, sex, ethnicity, employment status, source of participants, vaccination status, dominant variant, whether symptomatic, admitted to hospital, admitted to ICU, duration of long COVID.

b Day-level covariates: day of week, mean efforts in physical, cognitive, social or self-care activity, quality of previous night's sleep, mean activity efforts from previous day.

c Time-level covariates: time of day, efforts in physical, cognitive, social or self-care activity, whether slept or rested, activity efforts from previous time, lagged sleep or rest at previous time.

Small month-on-month improvements over the length of the study were seen in severity of fatigue (−0.04, −0.08 to −0.00, p = 0.04) and of dizziness (−0.04, −0.07 to −0.00, p = 0.03). Weekdays were associated with slightly worse severity across most symptoms, particularly cognitive dysfunction (0.3, 0.2–0.3, p < 0.001). Severity of many symptoms increased through the day, particularly fatigue (1.4, 1.0–1.7, p < 0.001) and cognitive dysfunction (0.7, 0.4–1.1, p < 0.001), whilst anxiety improved slightly. Need for rest or sleep during immediately prior to the EMA was associated with greater fatigue (0.6, 0.5–0.7, p < 0.001 and 1.0, 0.9–1.2, p < 0.001 respectively) and pain or discomfort (0.2, 0.1–0.3, p < 0.001 and 0.3, 0.2–0.5, p < 0.001 respectively), and rest with anxiety (0.1, 0.0–0.2, p = 0.04) and cognitive dysfunction (0.2, 0.1–0.3, p = 0.002). Rest 3 h previously was associated with slightly less cognitive dysfunction (−0.1, −0.2 to 0.0, p = 0.002) but sleep 3 h previously was associated with slightly worse depression scores (0.1, 0.0–0.2, p = 0.02).

### Activity efforts as predictors of symptom severity

The model incorporating delayed influences of activity efforts on subsequent symptoms through AR1 processes for both time-level and day-level mean symptom severities and activity efforts (Model 1) and the model incorporating a moving average (MA1) for symptoms severities (Model 3) both fitted substantially better than not accounting for time-dependency (Model 5), even allowing for the additional model complexity (Supplemental Table S1). The best fitting model incorporated the moving average MA1 process symptoms, with autoregressive processes of order 1 for the association between both day and time-level mean activity efforts and symptom severity (Model 3).

**Fig. 1:**
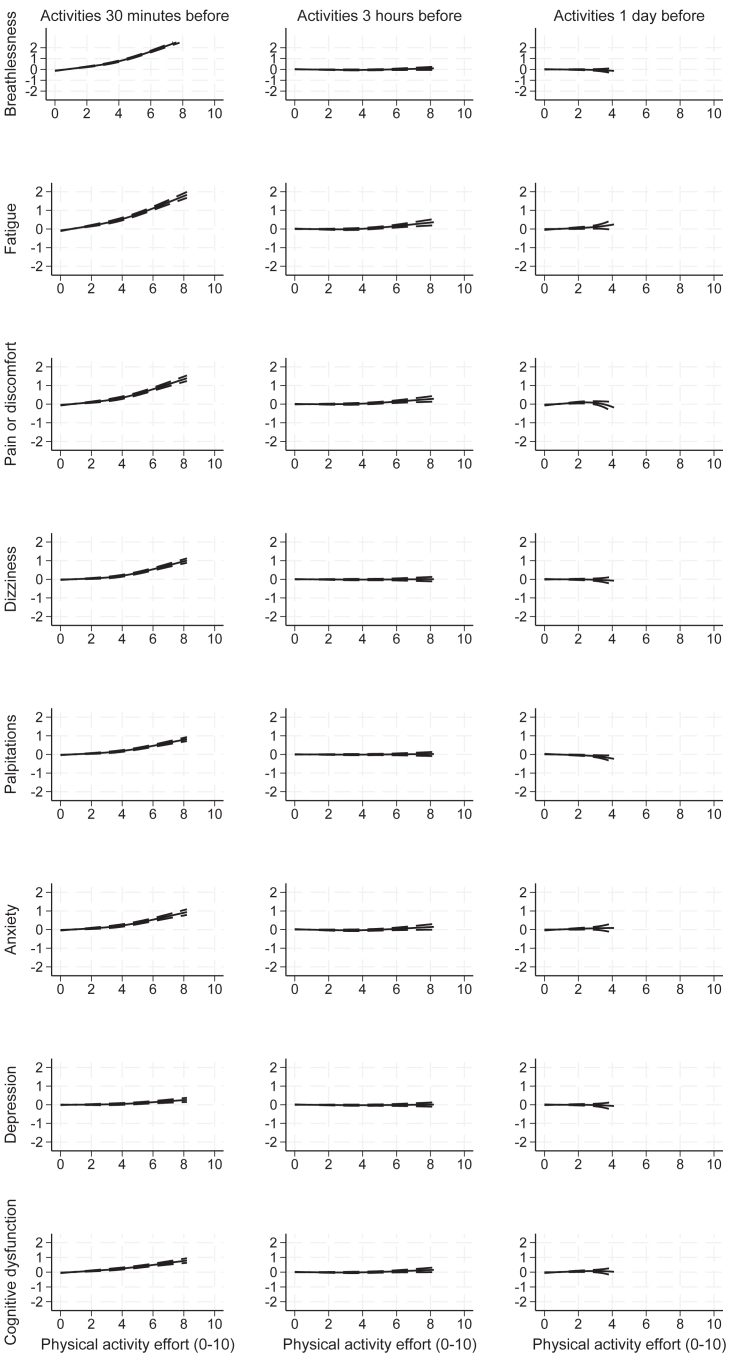
**Change in mean symptom severity scores associated with physical activity effort (0–10), by length of time-lag, with 95% credible intervals**. Activity effort is truncated at the 99th centile for presentation.

**Fig. 2:**
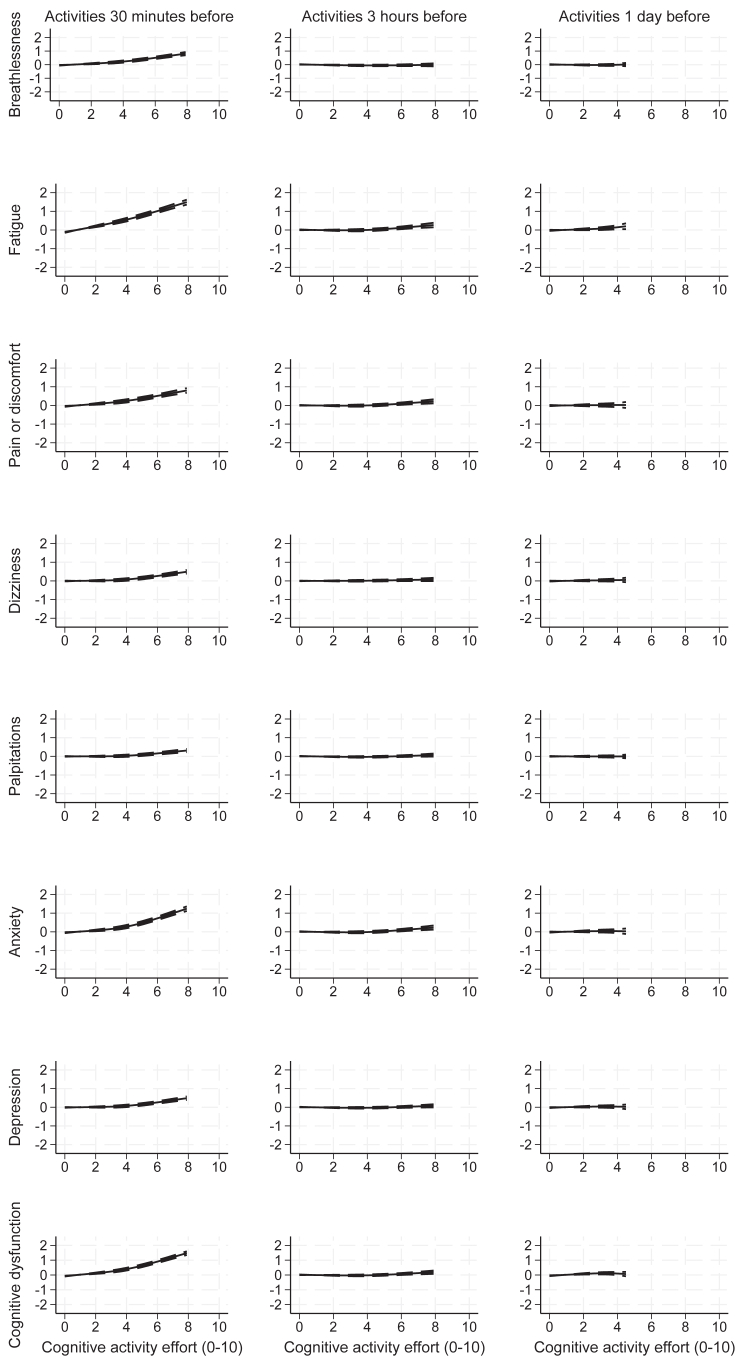
**Change in mean symptom severity scores associated with cognitive activity effort (0–10), by length of time-lag, with 95% credible intervals**. Activity effort is truncated at the 99th centile for presentation.

Fig. 1, Fig. 2 and Supplemental Figures S3 and S4 show the association between activity efforts (scored 0 to 10) and change in symptom severity scores (scored 0 to 10) for physical, cognitive, social and self-care activities respectively, for the best fitting model. The first column of each figure shows associations between symptoms and activities in the 30 min immediately prior to the EMA, the second column activities 3 h before (AR1 at time-level), and the third column 1 day before (AR1 at day-level).

All measured symptom severities increased after effort on all recorded activities in the 30 min prior to EMA (all p < 0.001) (Fig. 1, Fig. 2, Supplemental Figures S3 and S4, Supplemental Tables S3–S6). Effortful cognitive, social and self-care activities were all associated with increased symptom severity. High physical and cognitive efforts (exertions of 8/10) were associated with fatigue severity scores increased by 1.8/10 (95% CI: 1.6–1.9, p < 0.001) and 1.5/10 (1.4–1.7, p < 0.001) respectively. Severity of breathlessness was most strongly associated with physical activity, but increases in fatigue were as severe after cognitive, social and self-care activities as physical activities. Worse cognitive dysfunction was noted after cognitive activity, but was also apparent immediately after other activity. Symptom severity generally increased with increasing exertion, becoming more marked following efforts rated greater than 5 out of a possible 10.

There was evidence of small, delayed reactions to effort on all types of activities. Three hours after the high physical activity (exertion of 8/10), small, delayed increases were observed in severity of fatigue (0.3/10, 95% CI: 0.2–0.5, p < 0.001) and pain or discomfort (0.3, 0.1–0.4, p = 0.001). Three hours after the high cognitive effort (exertion of 8/10), further increases in fatigue (0.3, 0.2–0.4, p < 0.001), pain or discomfort (0.2, 0.1–0.3, p = 0.001), anxiety (0.2, 0.1–0.3, p < 0.001) and cognitive dysfunction (0.2, 0.1–0.3, p = 0.004) were observed. Similarly, greater fatigue emerged 3 h after high-effort (exertion of 8/10) social activity efforts (0.2, 0.0–0.5, p = 0.05). After high-effort self-care activities (exertion of 8/10), delayed fatigue (0.5, 0.3–0.7, p < 0.001), pain or discomfort (0.4, 0.2–0.6, p < 0.001), anxiety (0.3, 0.1–0.5, p = 0.02) and depression (0.2, 0.1–0.4, p = 0.02) were reported. However, delayed impacts of exertion emerging 3 h after the effort were small relative to increased symptoms within 30 min of the activity.

Efforts on some activities also had associated small, delayed changes in symptom severity emerging the following day. Effortful physical activity was associated with increased pain or discomfort and cognitive dysfunction the next day over most of the range, and decreased palpitations, though credible intervals are wide for higher efforts. Greater effort on cognitive activities was associated with more severe fatigue emerging and increased cognitive dysfunction reported the following day. Social activity the day before was associated with worse fatigue emerging the next day. Effortful self-care activity the day before was associated with increased fatigue and markedly increased severity of palpitations the following day, though mean efforts over each day were low and credible intervals were for all wide symptoms.

### Subgroup analyses

The best fitting model (Model 3) was run separately for the clinic and community (social media) samples, for men and women, and for younger (<50 years) and older participants (50+ years). Conclusions were broadly consistent across all subgroups (See Supplementary Material).

## Discussion

We have reported intensive longitudinal data from a large sample of people living with long COVID, prospectively monitoring exertion on physical, cognitive, social and self-care activities, and recording subsequent changes in severity of frequently reported symptoms. Our study represents the largest study of its kind in long COVID, using serial measurements of activities and symptoms throughout the day over several weeks. By recording severity of fatigue, breathlessness, pain and other symptoms every 3 h over multiple days, we were able to separately quantify the impact at the time of the activity and immediately afterwards, as well as longer-term onset the following day.

Cognitive, social, self-care and physical activities were all associated with subsequent increased severity of eight different symptoms revealing a probable common pathway as a potential therapeutic target. Symptom severity was elevated 30 min after activities, but there was evidence of only small delayed reactions after 3 h or the next day. Symptom severity increased through the day, but was lower at weekends. Symptom presentation varied across groups, with older people reporting worse breathlessness, pain or discomfort, and cognitive dysfunction, and women having more fatigue than men, but less dizziness, anxiety and depression. There was little difference in severity between broad ethnic groups, source of participants, or severity of initial infection. Participants in full-time employment had lower symptom scores, which may reflect their greater capacity to work, or socioeconomic inequalities. The clear patterns of symptom fluctuations support more targeted self-management.

A previous intensive longitudinal cohort demonstrated associations between physical activity and fatigue.^8^ Our findings, from a substantially larger study, also show how physical exertion is associated with increased severity, not only of fatigue and breathlessness shortly afterwards, but also a range of symptoms including dizziness and cognitive dysfunction, sometimes called “brain fog”. We also demonstrate how exertion in cognitive, social and self-care activities can all lead to worse severity across all symptoms. We found that increased severity was strongest soon after the activity, with symptom severity elevated 30 min after all categories of activity, but there was less evidence of delayed symptom response after 3 h or delayed to the next day. Resting appeared to be as a response to fatigue or pain, with rest or sleep immediately prior to the EMA associated with slightly worse symptoms, and little change 3 h later the same day.

Previous work has shown that symptoms of long COVID vary greatly between individuals but also fluctuate within the same individual.^5^^,^^6^ Episodes of exacerbated symptoms can follow relatively small exertions and with variable but potentially long duration of such episodes.^6^^,^^8^ Strong qualitative evidence for this exists from the lived experience of patients in long COVID clinics and living in the community. Our study adds to this body of evidence by quantifying the impact of a range of potential triggers, including cognitive tasks, and the extent to which some symptoms may not fully emerge until the next day.

The modelling shows that long COVID symptoms follow a pattern characterised by unexpected shifts in symptoms the day before affect the current symptoms, leading to periodic flare-ups in symptoms, typical of the moving average process. On top of this, the amount of effort exerted on physical, cognitive, social and self-care activities has an immediate impact on a wide range of symptoms, sometimes only emerging hours later or the next day, typical of an autoregressive process. Though our work offers only limited insight into discerning between proposed mechanisms of action, it is consistent with a common pathway, such as persistent viral infection leading to increased inflammatory response and symptom exacerbation, dysfunctional mitochondrial processes, or other alterations to skeletal muscle and its metabolism.^23^^,^^24^ Exploring how symptom fluctuations relate to potential mechanistic markers may be an important focus for future work.

In our work we have made the same modelling assumptions across all symptoms (e.g. AR1), but different symptoms may potentially be modelled better using different assumptions. This could add further insight to different mechanisms driving the severity of each symptom.

The response rate (82%) is likely to under-estimate the non-response in the community-based sample. The adherence rate (75%), whilst lower than some studies, is to be expected in people living with fatiguing chronic conditions like long COVID. Missing symptom data was generated from the posterior predictive distribution assuming MCAR, so may have under-estimated symptom severity if missingness is informative. With missing activity data, we assumed no exertion on that activity, which may have led to under-estimates of delayed symptom response.

Use of contemporaneous EMA methods reduces participant burden, leading to improved response rates and lower propensity to recall bias than larger questionnaires administered days or weeks later. However, symptom questions asked shortly after activity questions are still vulnerable to some potential recall bias. The short nature of the assessments also facilitates collection of the intensive longitudinal data required to investigate short-term fluctuations, as seen in long COVID.

A small proportion of participants reported pre-existing conditions that could account for the presence of some symptoms. Reviews of long COVID suggest comparison with healthy controls,^1^ different methods of recording symptoms,^3^ and whether their impacts on daily activities is taken into account,^3^ can influence estimated incidence rates. By contrast, we use internal controls to estimate within-person change in symptoms associated with exertion.

Randomised controlled trials of pacing are lacking, but our results are consistent with current recommendations for recovery and rehabilitation supporting the principle of individuals reducing symptom severity by planning activity levels to stay within their available energy envelope, which is the basis of the pacing approach to rehabilitation from long COVID.^25^^,^^26^ We also reveal that cognitive, social and self-care activities need to be included in this.

Long COVID research often focuses disproportionately on people who were hospitalized with the initial infection and could obtain a PCR or antibody test, and had predominantly respiratory symptoms.^27^^,^^28^ This leaves a substantial portion of people living with long COVID underrepresented, especially those with non-respiratory symptoms or those who were not hospitalized.^29^ Our study avoids this problem.

We had low minority ethnic representation, reflecting low representation across UK long COVID clinics generally.^30^ Analyses were therefore restricted to comparing White to all minority ethnic groups together, limiting scope for conclusions in this area. We found no evidence of major differences between white and minority ethnic groups, but other studies have revealed differences in symptom reporting and diagnosis,^31^ so larger samples may reveal important differences between minority ethnic groups in response to types of exertion.

There is also evidence that socioeconomic position is relevant to long COVID prevalence,^32^ intersecting with gender and occupation. Our findings show those in full-time employment experienced less severe symptoms than other participants, which may point to socioeconomic inequalities, and highlights the need for definitive research to clarify this relationship.

Participants from long COVID clinics may not be representative of all people living with long COVID in their comorbidities, symptom profiles, and symptom severities. This may potentially extend to the degree that symptoms fluctuate or the degree to which efforts worsen symptom severities, or avoidance of activities that lead to severe symptom exacerbation in this observational cohort. However, they should be representative of clinic populations. By contrast, our community-based sample maybe more self-selected, and not representative of the broad spectrum of people living with long COVID who are not in the care of specialist long COVID clinics. However, findings were broadly similar across both groups of participants. Similarly, though we did not adjust for menopausal status in women, results were similar above and below age 50 years.

In conclusion, this research makes an important contribution to the growing body of knowledge on long COVID. We found clear patterns of symptoms, which include both large immediate and small delayed responses to activity, including cognitive activity. This highlights the need for randomised controlled trials of targeted self-monitoring and management of activities and symptoms, and provides a potential framework to explore underlying mechanisms more effectively, to improve the quality of life for those living with this condition.

## Contributors

DBO’C and HD are responsible for the original concept. DCG, MM, JC, CR, IT, DBO’C and HD designed the study. DCG, NDB, BD, GM, MS, DBO’C, and HD acquired funding. MM, NDB, AB, JC, HED, JD, LE, PL, GM, WM, FR, JTS, HV, and TW conducted the investigation. DCG, MM, WM and HV wrote software. DCG, MM, and HV curated data. DCG designed and conducted statistical analysis. MM, DBO’C and HD administered the project. DBO’C and HD supervised staff. DCG and MM wrote the first draft. All authors critically reviewed and approved the manuscript.

All authors fully meet the ICMJE criteria for authorship. DCG, MM, HV, DBO’C and HD directly accessed and verified the underlying data reported in the manuscript. All authors had full access to all the data in the study and accept responsibility to submit for publication.

## Data sharing statement

Requests for de-identified participant data may be submitted to the authors. Applications, including a full research proposal and confirmation of appropriate ethical approval, will be evaluated for compatibility with general objectives, ethical approvals, existing informed consent received, and for potential overlap with ongoing work.

## Declaration of interests

This work is independent research funded by the National Institute for Health and Care Research (NIHR) (Long COVID grant, Ref: COV-LT2-0016). The views expressed in this publication are those of the authors and not necessarily those of NIHR or The Department of Health and Social Care. All authors were supported by funding from this source (DCG, MM, NDB, AB, JC, HED, JD, BD, LE, PL, GM, WM, CR, FR, JTS, MS, IT, HV, TW, DBO’C, HD).

DCG was additionally supported by funding from NHS England, NHS Improvement and NHS National Services Scotland. MM and HD are supported by the National Institute for Health and Care Research Exeter Biomedical Research Centre. JC is funded by the National Institute for Health and Care Research Oxford Health Biomedical Research Centre. BD is a member of the BioNTech Long COVID and vaccination review advisory board. MS was supported by funding from Research England Policy Support Fund and the Engineering and Physical Sciences Research Council, holds roles with the Oxford Handbook of Rehabilitation Medicine, Advances in Rehabilitation Science and Practice, Frontiers in Pain Research and the British Society of Physical and Rehabilitation Medicine.

This work was undertaken on ARC4, part of the High Performance Computing facilities at the University of Leeds, UK.

## Appendix A Supplementary data

Supplementary Figures and Tables

List of consortium members Lancet
