# Supplementary material for: Physical, cognitive, and social triggers of symptom fluctuations in people living with long COVID: an intensive longitudinal cohort study
Source: Lancet Reg Health Eur. 2024 Sep 20;46:101082. doi: 10.1016/j.lanepe.2024.101082 (PMC11458954; doi:10.1016/j.lanepe.2024.101082)
Supplement: Supplementary Figures and Tables [file mmc1.pdf]

Supplemental material

List of consortium members 2

Additional statistical methods 3

Supplemental Figure 1. Schedule of ecological momentary assessments recording activity efforts and symptom severities throughout the study 5

Supplemental Figure 2. Example ecological momentary assessment screenshots 6

Supplemental Figure 3. Change in mean symptom severity scores associated with social activity effort (0-10), by length of time-lag, with 95% credible intervals 7

Supplemental Figure 4. Change in mean symptom severity scores associated with self-care activity effort (0-10), by length of time-lag, with 95% credible intervals 8

Supplemental Table 1. Comparison of DIC values for fitted models 9

Supplemental Table 2. Characteristics of participants from long COVID clinic and community samples 10

Supplemental Table 3. Mean change in mean symptom severity scores associated with physical activity effort (0-10), by length of time-lag, with 95% credible intervals 11

Supplemental Table 4. Mean change in mean symptom severity scores associated with cognitive activity effort (0-10), by length of time-lag, with 95% credible intervals 12

Supplemental Table 5. Mean change in mean symptom severity scores associated with social activity effort (0-10), by length of time-lag, with 95% credible intervals 13

Supplemental Table 6. Mean change in mean symptom severity scores associated with self-care activity effort (0-10), by length of time-lag, with 95% credible intervals 14

References 15

List of consortium members

Nawar Bakerly, Kumaran Balasundaram, Megan Ball, Mauricio Barahona, Alexander Casson, Jonathan Clarke, Karen Cook, Rowena Cooper, Vasa Curcin, Julie Darbyshire, Helen E Davies, Helen Dawes, Simon de Lusignan, Brendan Delaney, Carlos Echevarria, Sarah Elkin, Ana Belen Espinosa Gonzalez, Rachael Evans, Sophie Evans, Zacchaeus Falope, Ben Glampson, Madeline Goodwin, Trish Greenhalgh, Darren C Greenwood, Stephen Halpin, Juliet Harris, Will Hinton, Mike Horton, Samantha Jones, Joseph Kwon, Cassie Lee, Ashliegh Lovett, Mae Mansoubi, Victoria Masey, Harsha Master, Erik Mayer, Bernardo Meza-Torres, Ruairidh Milne, Ghazala Mir, Jacqui Morris, Adam Mosley, Jordan Mullard, Daryl O'Connor, Rory O'Connor, Thomas Osborne, Amy Parkin, Stavros Petrou, Anton Pick, Denys Prociuk, Clare Rayner, Amy Rebane, Natalie Rogers, Janet T Scott, Manoj Sivan, Adam B Smith, Nikki Smith, Emma Tucker, Ian Tucker-Bell, Paul Williams, Darren Winch, Conor Wood.

Additional statistical methods

Statistical analysis

Multilevel multivariate vector autoregressive (VAR) modelling was used to quantify the associations between the activities reported in the EMA and subsequent symptom scores, taking account of the hierarchical data structure. Symptom scores were modelled as joint multivariate outcomes with all variances and covariances distinctly estimated.

All models included person-level covariates: age (as restricted cubic splines with knots at 40, 50 and 60 years), sex, ethnicity (white, minority ethnic), employment status (fulltime, part-time, not in paid employment), location (individual clinic or community setting), acute infection severity (asymptomatic, symptomatic), whether hospitalised, whether admitted to intensive care unit, dominant variant at time of infection (original, alpha, delta, omicron), vaccination status (completed two doses prior to infection), duration of long COVID symptoms. Models also included time-level covariates: time (9am, 12noon, 3pm, 6pm, 9pm) as restricted cubic splines with knots at 9am, 3pm and 9pm, exertions (physical, cognitive, social and self-care as restricted cubic splines with knots at 2, 5 and 8), and day-level covariates: rest during the day, sleep during the day, overnight sleep quality (<3, 3-4.9, 5-6.9, 7+, missing).

All covariates were mean-centred where appropriate. Absence of a reported activity was assumed to imply zero exertion on that activity. Missing response data were assumed missing at random (MAR) with values generated from the posterior predictive distribution.

Models of with different time-dependency between efforts and symptoms were compared using the Deviance Information Criterion (DIC), with lower DIC suggesting better fit and a difference >10 considered important[1,2]. Five sets of modelling assumptions were compared:

(1) Between-day and between-time components both modelled using autoregressive time-series of order 1 (AR1), with symptom severity at one time point estimated using symptoms and activity efforts at the previous time point (3 hours earlier), in addition to 30 minutes immediately prior to the EMA. Symptom severities on one day estimated using mean activity efforts and symptoms from the previous day (AR1). Activities at time points and days prior to data collection incorporated using latent variables. This modelled potentially delayed influences of trigger activities and efforts on subsequent symptoms to be quantified.

(2) As in the first model, but using the peak effort from the previous day rather than the mean to model where efforts exceeding a threshold were associated with worse symptoms.

(3) As in the first model, but using a moving average process of order 1 (MA1) for symptoms at both day and time levels, to model where symptom severity the previous day exceeds the norm in a way not fully absorbed that same day, and severity from 3 hours earlier persists to the next time point.

(4) As in the first model, but including cumulative efforts summed over all time points within the same day, to model where cumulating efforts were associated with worsening symptom scores.

(5) The above models were compared to a base model with no autoregressive relationship between time-level activity efforts and symptoms.

All stochastic parameters were given proper but minimally informative prior distributions, with symptom scores given a minimally informative Wishart prior. Three independent Markov chain Monte Carlo (MCMC) chains were used with dispersed initial values, adaptation and burn in of 10,000 iterations until convergence to stable distributions was achieved, followed by a further 20,000 updates for each chain. Adequate mixing and convergence were confirmed by trace plots and Brooks-Gelman-Rubin statistics[1], with the Monte Carlo error for each parameter being <5% of the sample standard deviation.

Confidence intervals for prevalence of symptoms and mean severity scores were derived from simple variance-components models allowing for variation at person, week, day and time-levels. Descriptive analysis was conducted using Stata 18[3]. MCMC models providing parameter estimates and 95% credible intervals were completed in JAGS 4.3.0[4] using the runjags package[5] from within R version 4.3.1[6] on the High Performance Computing facilities at the University of Leeds, UK. Two-sided p-values were derived from samples of the posterior distributions, where necessary.

Activity efforts as predictors of symptom severity

The model incorporating delayed influences of activity efforts on subsequent symptoms through AR1 processes for both time-level and day-level mean symptom severities and activity efforts (Model 1) and the model incorporating a moving average (MA1) for day-level symptoms severities (Model 3) both fitted substantially better than not accounting for time-dependency (Model 5), even allowing for the additional model complexity (Supplemental Table 1). The best fitting model incorporated the moving average MA1 process for symptoms, with autoregressive processes of order 1 for the association between both day and time-level mean activity efforts and symptom severity (Model 3).

Supplemental Figure 1. Schedule of ecological momentary assessments recording activity efforts and symptom severities throughout the study

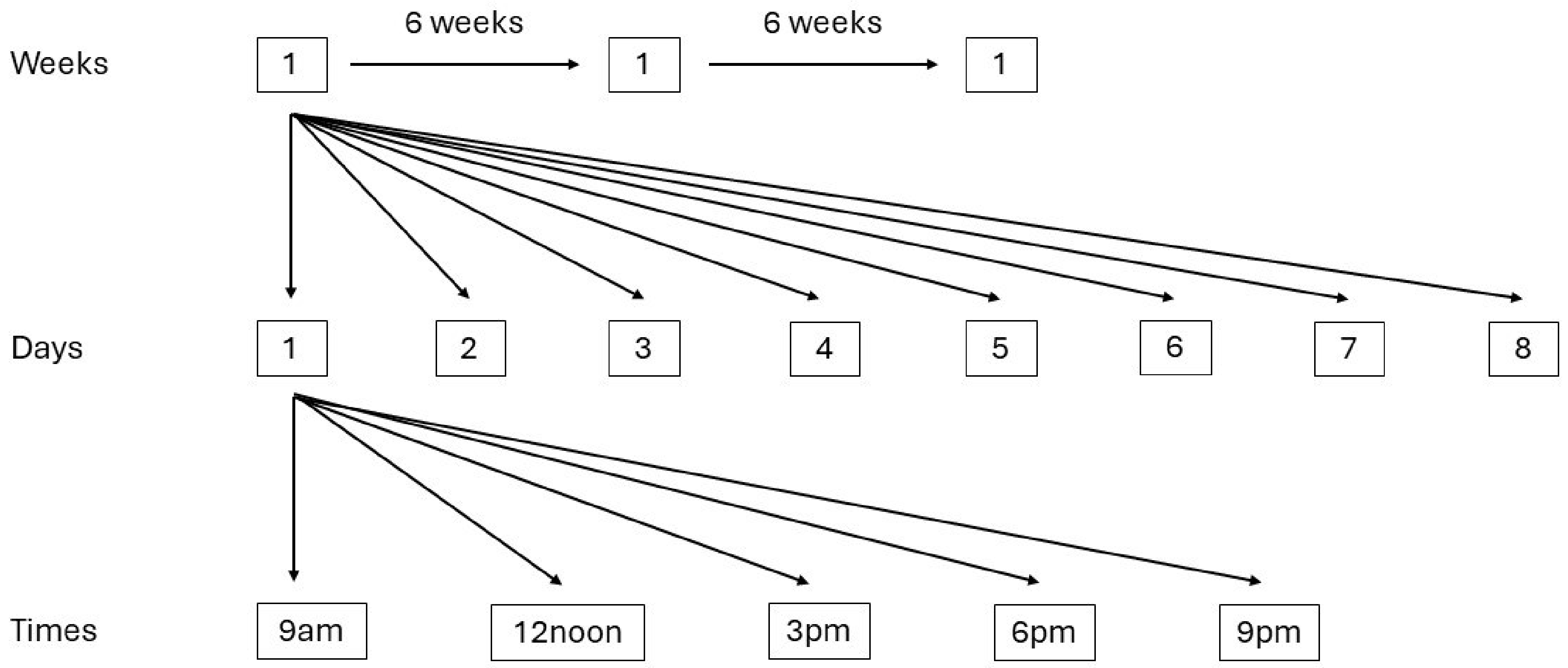

Supplemental Figure 2. Example ecological momentary assessment screenshots

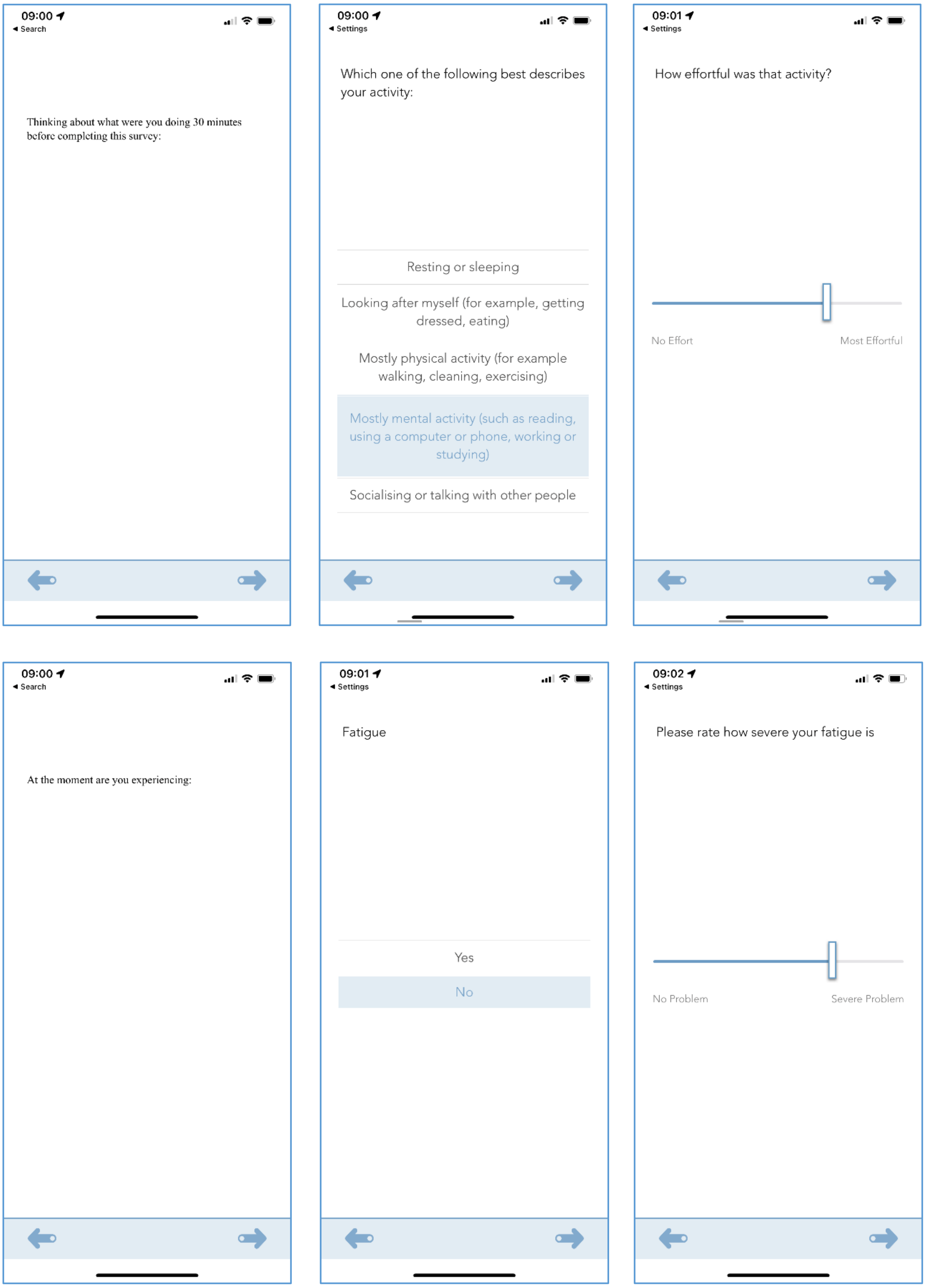

Supplemental Figure 3. Change in mean symptom severity scores associated with social activity effort (0-10), by length of time-lag, with 95% credible intervals

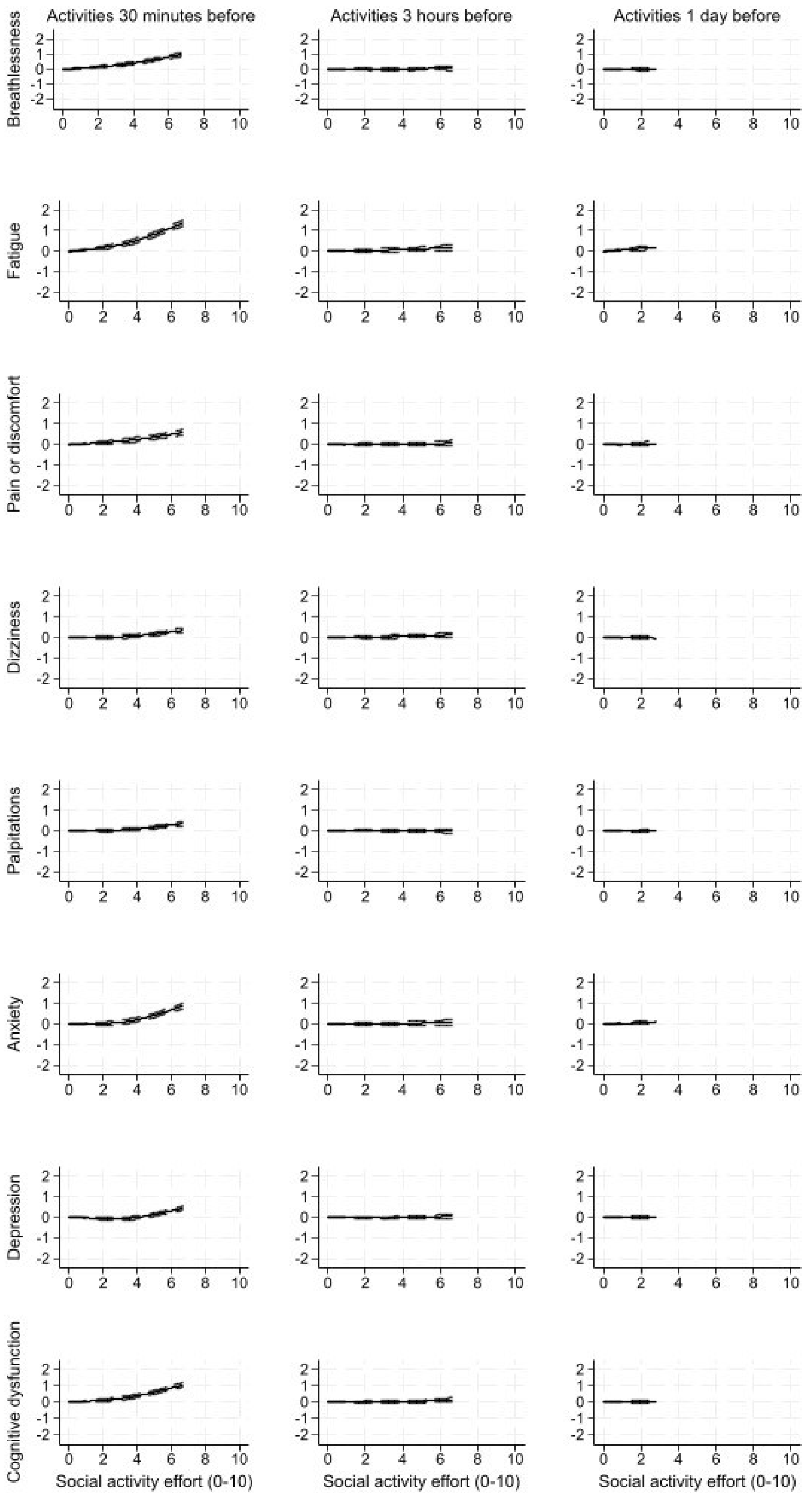

Activity effort truncated at 99th centile for presentation

Supplemental Figure 4. Change in mean symptom severity scores associated with self-care activity effort (0-10), by length of time-lag, with 95% credible intervals

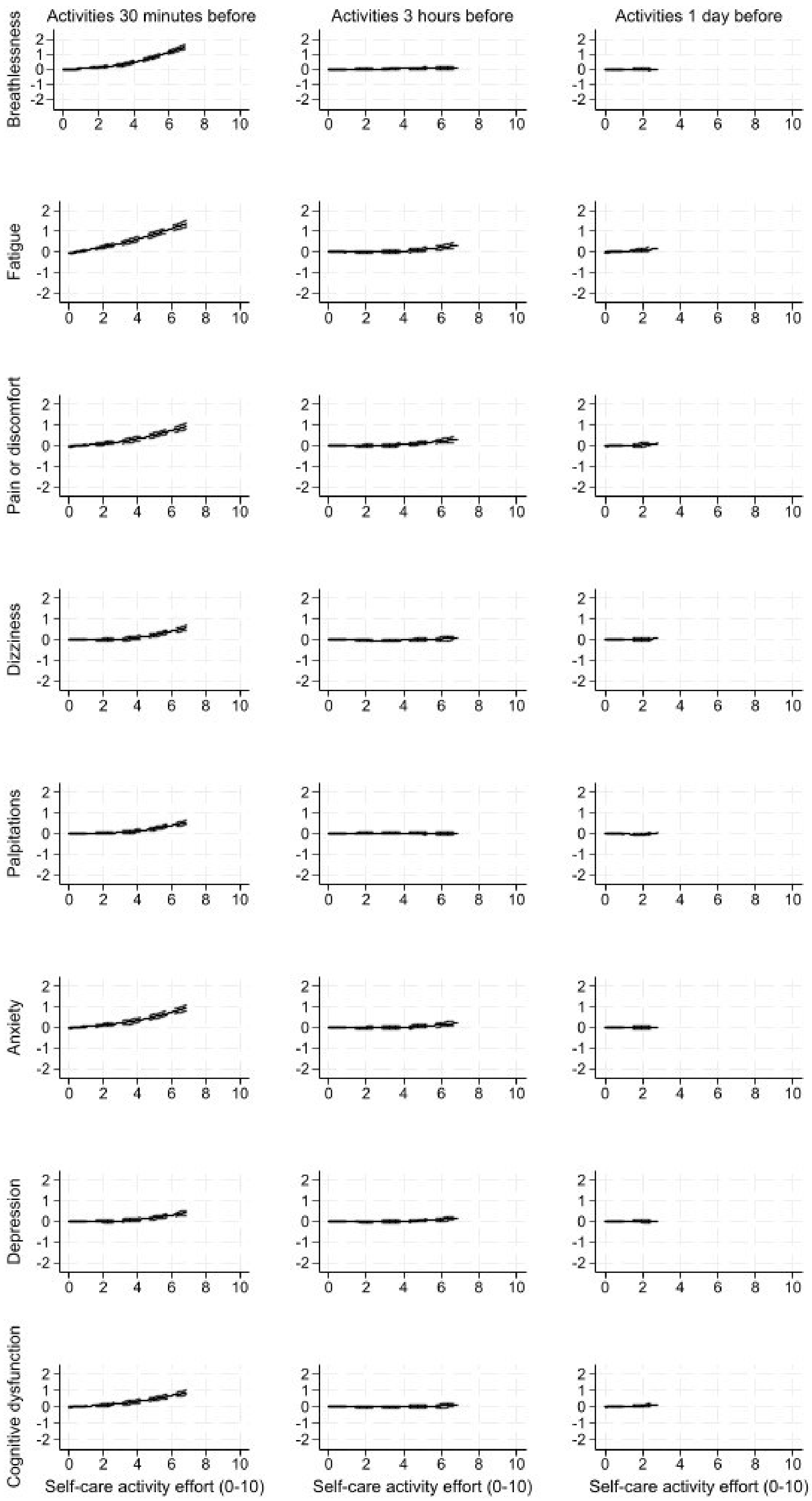

Activity effort truncated at 99th centile for presentation

Supplemental Table 1. Comparison of DIC values for fitted models

	Model	Deviance	pD	DIC
(1)	Time-level symptom severities depend on day-level mean symptoms, activity efforts 30 minutes immediately preceding EMA, time-level covariates, and:<br>AR1 for symptoms from previous time point<br>AR1 for activity efforts from previous time<br><br>Day-level mean symptom severities depend on participant, day-level covariates, and:<br>AR1 for mean symptoms severities from the previous day<br>AR1 for mean activity efforts from the previous day<br><br>Participant mean symptom severities depend on participant-level covariates.	712768	253562	966330
(2)	As in Model 1, but using the peak activity effort from the previous day instead of the mean.	712753	263881	976634
(3)	As in Model 1, but using a moving average model of order 1 (MA1) over both day and time-level symptom severities.	710448	198903	909351
(4)	As in Model 1, but including cumulative efforts summed over all time points within the same day	713109	276845	989955
(5)	As in Model 1, but with no relationship between symptom severities and efforts at previous time points	712781	260663	973444

Supplemental Table 2. Characteristics of participants from long COVID clinic and community samples

	Long COVID clinic patients (n=273)	Community-based (n=103)	Total (n=376)
Mean age (years)(SD)	46 (12)	49 (10)	47 (11)
Female gender (%)	192 (70%)	82 (80%)	274 (73%)
Ethnicity:			
White (%)	236 (86%)	90 (87%)	326 (87%)
Black (%)	0 (0%)	2 (2%)	2 (1%)
Asian (%)	16 (6%)	1 (<1%)	17 (5%)
Mixed/other (%)	21 (8%)	10 (10%)	31 (8%)
Employment status:			
Full-time (%)	141 (52%)	31 (30%)	172 (46%)
Part-time (%)	48 (18%)	22 (21%)	70 (19%)
Self-employed (%)	16 (6%)	5 (5%)	21 (6%)
Not in paid employment (%)	67 (25%)	45 (44%)	112 (30%)
Not recorded (%)	1 (<1%)	0 (0%)	1 (<1%)
Pre-existing comorbidities:			
Allergies or autoimmune conditions (%)	36 (13%)	22 (21%)	58 (15%)
Other respiratory conditions (%)	2 (1%)	0 (0%)	2 (1%)
Other inflammatory conditions (%)	7 (3%)	0 (0%)	7 (2%)
Hypertension (%)	7 (3%)	2 (2%)	9 (2%)
Hypotension (%)	0 (0%)	0 (0%)	0 (0%)
Other heart conditions (%)	5 (2%)	3 (3%)	8 (2%)
Type 2 diabetes mellitus (%)	6 (2%)	0 (0%)	6 (2%)
Mental health condition (%)	42 (15%)	11 (11%)	53 (14%)
Completed vaccinations before initial infection (%)	146 (53%)	48 (47%)	194 (52%)
Dominant variant at time of infection:			
Original (%)	91 (33%)	44 (43%)	135 (36%)
Alpha (%)	25 (9%)	9 (9%)	34 (9%)
Delta (%)	68 (25%)	16 (16%)	84 (22%)
Omicron (%)	89 (33%)	34 (33%)	123 (33%)
Positive SARS-CoV-2 test (%)	251 (92%)	84 (82%)	335 (89%)
Asymptomatic with initial infection (%)	10 (4%)	1 (<1%)	11 (3%)
Admitted to hospital with initial infection (%)	28 (10%)	5 (5%)	33 (9%)
Admitted to intensive care with initial infection (%)	10 (4%)	1 (<1%)	11 (3%)
Median duration of long COVID (IQR) (months)	15 (9, 24)	24 (14, 36)	17 (10, 26)
Clinic location:			
Birmingham (%)	18 (7%)	-	-
Cardiff (%)	56 (21%)	-	-
Hertfordshire (%)	31 (11%)	-	-
NHS Highland (%)	8 (3%)	-	-
Imperial College London (%)	11 (4%)	-	-
Leeds (%)	11 (4%)	-	-
Leicester (%)	5 (2%)	-	-
Newcastle (%)	20 (7%)	-	-
Oxford (%)	76 (28%)	-	-
Salford (%)	37 (14%)	-	-

Supplemental Table 3. Mean change in mean symptom severity scores associated with physical activity effort (0-10), by length of time-lag, with 95% credible intervals

Physical activity effort (0-10)	Breathless-ness	Fatigue	Pain / discomfort	Dizziness	Palpitations	Anxiety	Depression	Cognitive dysfunction
30 minutes before								
0	-	-	-	-	-	-	-	-
2	0·2 (0·2, 0·3)	0·2 (0·1, 0·2)	0·1 (0·1, 0·1)	0·0 (0·0, 0·1)	0·1 (0·0, 0·1)	0·1 (0·0, 0·1)	0·0 (0·0, 0·0)	0·1 (0·1, 0·1)
4	0·8 (0·7, 0·8)	0·5 (0·5, 0·6)	0·3 (0·3, 0·4)	0·2 (0·1, 0·2)	0·2 (0·1, 0·2)	0·2 (0·2, 0·3)	0·0 (0·0, 0·1)	0·2 (0·2, 0·3)
6	1·6 (1·6, 1·7)	1·1 (1·0, 1·2)	0·8 (0·7, 0·9)	0·5 (0·5, 0·6)	0·5 (0·4, 0·5)	0·5 (0·4, 0·6)	0·1 (0·1, 0·2)	0·5 (0·4, 0·6)
8	2·7 (2·6, 2·8)	1·8 (1·6, 1·9)	1·3 (1·2, 1·5)	1·0 (0·8, 1·1)	0·8 (0·7, 0·9)	0·9 (0·8, 1·0)	0·2 (0·1, 0·4)	0·8 (0·6, 0·9)
3 hours before								
0	-	-	-	-	-	-	-	-
2	0·0 (-0·1, 0·0)	0·0 (0·0, 0·0)	0·0 (0·0, 0·0)	0·0 (0·0, 0·0)	0·0 (0·0, 0·0)	0·0 (0·0, 0·0)	0·0 (0·0, 0·0)	0·0 (0·0, 0·0)
4	0·0 (-0·1, 0·0)	0·0 (0·0, 0·1)	0·0 (0·0, 0·1)	0·0 (-0·1, 0·0)	0·0 (0·0, 0·0)	0·0 (-0·1, 0·0)	0·0 (-0·1, 0·0)	0·0 (-0·1, 0·0)
6	0·0 (-0·1, 0·1)	0·2 (0·1, 0·2)	0·1 (0·1, 0·2)	0·0 (-0·1, 0·0)	0·0 (0·0, 0·1)	0·0 (0·0, 0·1)	0·0 (-0·1, 0·0)	0·1 (0·0, 0·1)
8	0·1 (0·0, 0·2)	0·3 (0·2, 0·5)	0·3 (0·1, 0·4)	0·0 (-0·1, 0·1)	0·0 (-0·1, 0·1)	0·1 (0·0, 0·3)	0·0 (-0·1, 0·1)	0·1 (0·0, 0·3)
1 day before								
0	-	-	-	-	-	-	-	-
2	0·0 (-0·1, 0·0)	0·0 (0·0, 0·1)	0·1 (0·0, 0·1)	0·0 (0·0, 0·0)	0·0 (-0·1, 0·0)	0·0 (0·0, 0·1)	0·0 (0·0, 0·0)	0·1 (0·0, 0·1)
4	-0·1 (-0·4, 0·1)	0·2 (0·0, 0·5)	-0·1 (-0·4, 0·1)	-0·1 (-0·3, 0·1)	-0·2 (-0·4, 0·0)	0·1 (-0·2, 0·3)	-0·1 (-0·3, 0·1)	0·0 (-0·2, 0·3)

Activity effort is truncated at the 99th centile for presentation

Supplemental Table 4. Mean change in mean symptom severity scores associated with cognitive activity effort (0-10), by length of time-lag, with 95% credible intervals

Cognitive activity effort (0-10)	Breathless-ness	Fatigue	Pain / discomfort	Dizziness	Palpitations	Anxiety	Depression	Cognitive dysfunction
30 minutes before								
0	-	-	-	-	-	-	-	-
2	0·1 (0·0, 0·1)	0·2 (0·2, 0·2)	0·1 (0·0, 0·1)	0·0 (0·0, 0·0)	0·0 (0·0, 0·0)	0·1 (0·0, 0·1)	0·0 (0·0, 0·0)	0·1 (0·1, 0·2)
4	0·2 (0·1, 0·3)	0·6 (0·5, 0·6)	0·2 (0·2, 0·3)	0·1 (0·0, 0·1)	0·0 (0·0, 0·1)	0·3 (0·2, 0·4)	0·1 (0·0, 0·1)	0·4 (0·3, 0·5)
6	0·5 (0·4, 0·6)	1·0 (0·9, 1·1)	0·5 (0·4, 0·6)	0·3 (0·2, 0·3)	0·2 (0·1, 0·2)	0·7 (0·6, 0·8)	0·3 (0·2, 0·3)	0·9 (0·8, 1·0)
8	0·8 (0·7, 0·9)	1·5 (1·4, 1·7)	0·8 (0·7, 0·9)	0·5 (0·4, 0·6)	0·3 (0·2, 0·4)	1·3 (1·1, 1·4)	0·5 (0·4, 0·6)	1·5 (1·4, 1·6)
3 hours before								
0	-	-	-	-	-	-	-	-
2	0·0 (-0·1, 0·0)	0·0 (0·0, 0·0)	0·0 (0·0, 0·0)	0·0 (0·0, 0·0)	0·0 (0·0, 0·0)	0·0 (0·0, 0·0)	0·0 (0·0, 0·0)	0·0 (-0·1, 0·0)
4	-0·1 (-0·1, 0·0)	0·0 (-0·1, 0·1)	0·0 (-0·1, 0·1)	0·0 (0·0, 0·1)	0·0 (-0·1, 0·0)	0·0 (-0·1, 0·0)	0·0 (-0·1, 0·0)	0·0 (-0·1, 0·0)
6	0·0 (-0·1, 0·0)	0·1 (0·0, 0·2)	0·1 (0·0, 0·2)	0·0 (0·0, 0·1)	0·0 (0·0, 0·1)	0·1 (0·0, 0·2)	0·0 (0·0, 0·1)	0·1 (0·0, 0·1)
8	0·0 (-0·1, 0·1)	0·3 (0·2, 0·4)	0·2 (0·1, 0·3)	0·1 (0·0, 0·2)	0·1 (0·0, 0·2)	0·2 (0·1, 0·3)	0·1 (0·0, 0·2)	0·2 (0·1, 0·3)
1 day before								
0	-	-	-	-	-	-	-	-
2	0·0 (-0·1, 0·0)	0·0 (0·0, 0·1)	0·0 (0·0, 0·1)	0·0 (0·0, 0·1)	0·0 (0·0, 0·0)	0·0 (0·0, 0·1)	0·0 (0·0, 0·1)	0·1 (0·0, 0·1)
4	0·0 (-0·1, 0·1)	0·1 (0·0, 0·3)	0·0 (-0·1, 0·1)	0·0 (0·0, 0·1)	0·0 (-0·1, 0·1)	0·0 (-0·1, 0·1)	0·0 (-0·1, 0·1)	0·1 (0·0, 0·2)

Activity effort is truncated at the 99th centile for presentation

Supplemental Table 5. Mean change in mean symptom severity scores associated with social activity effort (0-10), by length of time-lag, with 95% credible intervals

Social activity effort (0-10)	Breathless-ness	Fatigue	Pain / discomfort	Dizziness	Palpitations	Anxiety	Depression	Cognitive dysfunction
30 minutes before								
0	-	-	-	-	-	-	-	-
2	0·1 (0·1, 0·2)	0·2 (0·1, 0·2)	0·1 (0·0, 0·2)	0·0 (0·0, 0·1)	0·0 (0·0, 0·1)	0·0 (0·0, 0·1)	-0·1 (-0·1, 0·0)	0·1 (0·0, 0·2)
4	0·4 (0·3, 0·5)	0·5 (0·4, 0·6)	0·2 (0·1, 0·3)	0·1 (0·0, 0·2)	0·1 (0·0, 0·2)	0·2 (0·1, 0·3)	0·0 (-0·1, 0·1)	0·4 (0·3, 0·5)
6	0·8 (0·7, 0·9)	1·1 (1·0, 1·3)	0·5 (0·4, 0·6)	0·3 (0·2, 0·4)	0·3 (0·2, 0·4)	0·7 (0·6, 0·8)	0·3 (0·2, 0·4)	0·9 (0·7, 1·0)
8	1·3 (1·1, 1·4)	1·8 (1·6, 2·0)	0·8 (0·6, 1·0)	0·5 (0·3, 0·6)	0·5 (0·4, 0·6)	1·3 (1·1, 1·4)	0·7 (0·5, 0·8)	1·4 (1·2, 1·6)
3 hours before								
0	-	-	-	-	-	-	-	-
2	0·0 (-0·1, 0·1)	0·0 (0·0, 0·1)	0·0 (-0·1, 0·1)	0·0 (0·0, 0·1)	0·0 (0·0, 0·1)	0·0 (-0·1, 0·1)	0·0 (-0·1, 0·0)	0·0 (-0·1, 0·1)
4	0·0 (-0·1, 0·1)	0·1 (0·0, 0·2)	0·0 (-0·1, 0·1)	0·1 (0·0, 0·1)	0·0 (0·0, 0·1)	0·0 (-0·1, 0·1)	0·0 (-0·1, 0·0)	0·0 (-0·1, 0·1)
6	0·0 (-0·1, 0·2)	0·1 (0·0, 0·3)	0·0 (-0·1, 0·2)	0·1 (0·0, 0·2)	0·0 (-0·1, 0·1)	0·1 (-0·1, 0·2)	0·0 (-0·1, 0·1)	0·1 (0·0, 0·2)
8	0·1 (-0·1, 0·3)	0·2 (0·0, 0·5)	0·1 (-0·1, 0·3)	0·2 (0·0, 0·3)	-0·1 (-0·2, 0·1)	0·1 (-0·1, 0·3)	0·1 (-0·1, 0·3)	0·2 (0·0, 0·4)
1 day before								
0	-	-	-	-	-	-	-	-
2	0·0 (-0·1, 0·1)	0·1 (0·0, 0·2)	0·0 (-0·1, 0·1)	0·0 (-0·1, 0·1)	0·0 (-0·1, 0·0)	0·1 (0·0, 0·1)	0·0 (-0·1, 0·0)	0·0 (0·0, 0·1)
4	0·1 (-0·3, 0·4)	0·2 (-0·2, 0·6)	0·1 (-0·3, 0·4)	-0·1 (-0·4, 0·1)	0·1 (-0·2, 0·3)	0·3 (0·0, 0·6)	0·1 (-0·2, 0·3)	0·1 (-0·3, 0·4)

Activity effort is truncated at the 99th centile for presentation

Supplemental Table 6. Mean change in mean symptom severity scores associated with self-care activity effort (0-10), by length of time-lag, with 95% credible intervals

Self-care activity effort (0-10)	Breathless-ness	Fatigue	Pain / discomfort	Dizziness	Palpitations	Anxiety	Depression	Cognitive dysfunction
30 minutes before								
0	-	-	-	-	-	-	-	-
2	0·1 (0·1, 0·2)	0·2 (0·2, 0·3)	0·1 (0·0, 0·2)	0·0 (-0·1, 0·0)	0·0 (0·0, 0·1)	0·1 (0·0, 0·2)	0·0 (0·0, 0·1)	0·1 (0·0, 0·2)
4	0·5 (0·4, 0·6)	0·6 (0·5, 0·7)	0·3 (0·2, 0·4)	0·1 (0·0, 0·2)	0·1 (0·0, 0·2)	0·3 (0·2, 0·5)	0·1 (0·0, 0·2)	0·3 (0·2, 0·4)
6	1·1 (1·0, 1·3)	1·1 (1·0, 1·3)	0·7 (0·6, 0·9)	0·4 (0·3, 0·5)	0·4 (0·3, 0·5)	0·7 (0·6, 0·9)	0·3 (0·2, 0·4)	0·7 (0·6, 0·8)
8	1·9 (1·7, 2·1)	1·7 (1·5, 1·9)	1·2 (1·0, 1·4)	0·8 (0·6, 0·9)	0·7 (0·6, 0·9)	1·2 (1·0, 1·4)	0·6 (0·4, 0·7)	1·1 (0·9, 1·3)
3 hours before								
0	-	-	-	-	-	-	-	-
2	0·0 (0·0, 0·1)	0·0 (-0·1, 0·0)	0·0 (0·0, 0·1)	0·0 (-0·1, 0·0)	0·0 (0·0, 0·1)	0·0 (-0·1, 0·0)	0·0 (-0·1, 0·0)	0·0 (-0·1, 0·0)
4	0·0 (0·0, 0·1)	0·0 (0·0, 0·1)	0·1 (0·0, 0·1)	0·0 (-0·1, 0·0)	0·0 (0·0, 0·1)	0·0 (-0·1, 0·1)	0·0 (-0·1, 0·1)	0·0 (-0·1, 0·1)
6	0·1 (0·0, 0·2)	0·2 (0·1, 0·3)	0·2 (0·1, 0·3)	0·0 (0·0, 0·1)	0·0 (-0·1, 0·1)	0·1 (0·0, 0·2)	0·1 (0·0, 0·2)	0·1 (0·0, 0·2)
8	0·1 (-0·1, 0·3)	0·5 (0·3, 0·7)	0·4 (0·2, 0·6)	0·1 (0·0, 0·3)	0·0 (-0·2, 0·1)	0·3 (0·1, 0·5)	0·2 (0·1, 0·4)	0·2 (0·0, 0·4)
1 day before								
0	-	-	-	-	-	-	-	-
2	0·0 (-0·1, 0·1)	0·1 (0·0, 0·2)	0·1 (0·0, 0·1)	0·0 (0·0, 0·1)	0·0 (-0·1, 0·0)	0·0 (-0·1, 0·1)	0·0 (0·0, 0·1)	0·1 (0·0, 0·1)
4	0·0 (-0·4, 0·5)	0·4 (-0·1, 0·9)	0·4 (-0·1, 0·9)	0·2 (-0·1, 0·6)	0·6 (0·3, 0·9)	0·0 (-0·5, 0·5)	-0·3 (-0·7, 0·1)	0·5 (0·0, 1·0)

Activity effort is truncated at the 99th centile for presentation

References

1. Congdon P. Bayesian Statistical Modelling. 2nd ed. Chichester: Wiley; 2006.
2. Lunn DJ, C.; Best, N.;Thomas, A.; Spiegelhalter, D. The BUGS book: A practical introduction to Bayesian analysis. London: CRC Press; 2013.
3. StataCorp. Stata statistical software: Release 18. College Station, TX: StataCorp LLC; 2023.
4. Plummer M. JAGS version 4.3.0 user manual. 28/06/2017 ed; 2017.
5. Denwood MJ. runjags: An R Package Providing Interface Utilities, Model Templates, Parallel Computing Methods and Additional Distributions for MCMC Models in JAGS. *Journal of Statistical Software* 2016; **71**(9): 1 - 25.
6. R Core Team. R: A Language and Environment for Statistical Computing. 2023. https://www.R-project.org.
