# Supplementary material for: Physical, cognitive, and social triggers of symptom fluctuations in people living with long COVID: an intensive longitudinal cohort study
Source: Lancet Reg Health Eur. 2024 Sep 20;46:101082. doi: 10.1016/j.lanepe.2024.101082 (PMC11458954; doi:10.1016/j.lanepe.2024.101082)
Supplement: List of consortium members Lancet [file mmc2.docx]

**List of consortium members**

| Nawar D | Bakerly |
| --- | --- |
| Kumaran | Balasundaram |
| Megan | Ball |
| Mauricio | Barahona |
| Alexander | Casson |
| Jonathan | Clarke |
| Karen | Cook |
| Rowena | Cooper |
| Vasa | Curcin |
| Julie | Darbyshire |
| Helen E | Davies |
| Helen | Dawes |
| Simon | de Lusignan |
| Brendan | Delaney |
| Carlos | Echevarria |
| Sarah | Elkin |
| Ana Belen | Espinosa Gonzalez |
| Rachael | Evans |
| Sophie | Evans |
| Zacchaeus | Falope |
| Ben | Glampson |
| Madeline | Goodwin |
| Trish | Greenhalgh |
| Darren C | Greenwood |
| Stephen | Halpin |
| Juliet | Harris |
| Will | Hinton |
| Mike | Horton |
| Samantha | Jones |
| Joseph | Kwon |
| Cassie | Lee |
| Ashliegh | Lovett |
| Mae | Mansoubi |
| Victoria | Masey |
| Harsha | Master |
| Erik | Mayer |
| Bernardo | Meza-Torres |
| Ruairidh | Milne |
| Ghazala | Mir |
| Jacqui | Morris |
| Adam | Mosley |
| Jordan | Mullard |
| Daryl | O'Connor |
| Rory | O'Connor |
| Thomas | Osborne |
| Amy | Parkin |
| Stavros | Petrou |
| Anton | Pick |
| Denys | Prociuk |
| Clare | Rayner |
| Amy | Rebane |
| Natalie | Rogers |
| Janet T | Scott |
| Manoj | Sivan |
| Adam B | Smith |
| Nikki | Smith |
| Emma | Tucker |
| Ian | Tucker-Bell |
| Paul | Williams |
| Darren | Winch |
| Conor | Wood |
